# Secondary metabolite profiles and anti‐SARS‐CoV‐2 activity of ethanolic extracts from nine genotypes of *Cannabis sativa* L.

**DOI:** 10.1002/ardp.202400607

**Published:** 2024-11-14

**Authors:** Ermin Schadich, Dominika Kaczorová, Tibor Béres, Petr Džubák, Marián Hajdúch, Petr Tarkowski, Sanja Ćavar Zeljković

**Affiliations:** ^1^ Institute of Molecular and Translational Medicine, Faculty of Medicine and Dentistry Palacký University Olomouc Czech Republic; ^2^ Czech Advanced Technology and Research Institute (CATRIN) Palacký University Olomouc Czech Republic; ^3^ Department of Genetic Resources for Vegetables, Medicinal and Special Plants Crop Research Institute Olomouc Czech Republic; ^4^ Department of Biochemistry, Faculty of Science Palacký University Olomouc Czech Republic

**Keywords:** antiviral activity, *Cannabis sativa*, chemical composition, phytocannabinoids, SARS‐CoV‐2

## Abstract

This study deals with the comprehensive phytochemical composition and antiviral activity against SARS‐CoV‐2 of acidic (non‐decarboxylated) and neutral (decarboxylated) ethanolic extracts from seven high‐cannabidiol (CBD) and two high‐Δ^9^‐tetrahydrocannabinol (Δ^9^‐THC) *Cannabis sativa* L. genotypes. Their secondary metabolite profiles, phytocannabinoid, terpenoid, and phenolic, were determined by LC‐UV, GC‐MS, and LC‐MS/MS analyses, respectively. All three secondary metabolite profiles, cannabinoid, terpenoid, and phenolic, varied significantly among cannabinoid extracts of different genotypes. The dose–response analyses of their antiviral activity against SARS‐CoV‐2 showed that only the single predominant phytocannabinoids (CBD or THC) of the neutral extracts exhibited antiviral activity (all IC_50_ < 10.0 μM). The correlation matrix between phytoconstituent levels and antiviral activity revealed that the phenolic acids, salicylic acid and its glucoside, chlorogenic acid, and ferulic acid, and two flavonoids, abietin, and luteolin, in different cannabinoid extracts from high‐CBD genotypes are implicated in the genotype‐distinct antagonistic effects on the predominant phytocannabinoid. On the other hand, these analyses also suggested that the other phytocannabinoids and the flavonoid orientin can enrich the extract's pharmacological profiles. Thus, further preclinical studies on cannabinoid extract formulations with adjusted non‐phytocannabinoid compositions are warranted to develop supplementary antiviral treatments.

## Graphical abstract

Secondary metabolite profiles, phytocannabinoid, phenolic, and terpenoid profiles, and antiviral activity against SARS‐CoV‐2 of ethanolic extracts from nine *Cannabis sativa* L. genotypes were investigated, identifying distinct phytocannabinoid and phenolic components associated with the antiviral property and pharmacological profiles of the effective cannabinoid extracts. These cannabinoid extracts could be used as antiviral agents in the development of pharmaceutical dosage forms.

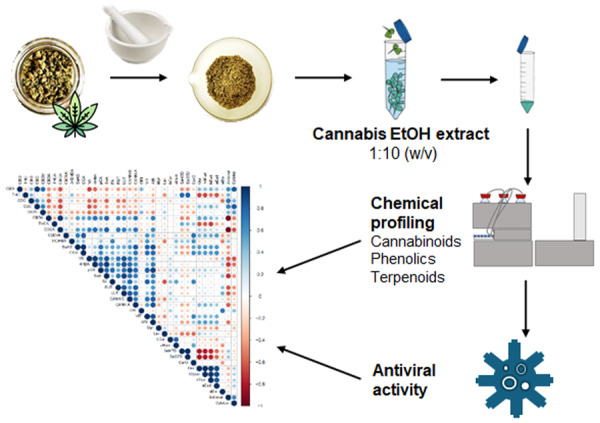

## 1 INTRODUCTION

The emergency of respiratory syndrome coronavirus 2 (SARS‐CoV‐2), a novel human virus from the Coronaviridae family, has caused the outbreak of highly contagious coronavirus disease (COVID‐19) at the end of 2019.^[^ ^1^ ^]^ SARS‐CoV‐2 has distinct virulence factors that are implicated in cellular tropism in the respiratory tract and very high virion transmissibility.^[^ ^2^ ^]^ Its rapid spread brought up 3 years of lasting COVID‐19 pandemic with fatal human and economic tolls. A comprehensive list of more than 700 compounds with anti‐SARS‐CoV‐2 activities in preclinical and/or clinical trials was published lately.^[^ ^3^ ^]^ One formidable approach could be to complement the currently used antivirotics and vaccines with novel effective agents like compounds of natural origin with less severe adverse effects.^[^ ^4^ ^]^ For such an approach, the bioactive compounds from plants like cannabis are good candidates.^[^ ^5^ ^]^

Cannabis was used for medicinal purposes to treat malaria, rabies, tetanus, rheumatism, and other diseases in countries such as China, Egypt, and India in 2000, 1550, and 100 B.C., respectively.^[^ ^6^ ^]^ Currently, it is accepted that the genus *Cannabis* is monotypic and consists only of a single species *Cannabis sativa* L.^[^ ^7^ ^]^ Cannabis flowers and leaves contain around 180 phytocannabinoids, compounds with a 21‐carbon terpene‐phenolic skeleton, and 320 other secondary metabolites identified as different terpenes and phenolics.^[^ ^8^ ^]^ Their pharmacological properties are attributable to the three most abundant bioactive phytocannabinoids that are naturally present as their corresponding carboxylic acid, including Δ^9^‐tetrahydrocannabinol (Δ9‐THC), cannabidiol (CBD), and cannabichromene (CBC). Previous in vitro and in vivo studies showed that both single phytocannabinoids and plant extracts have different antioxidant, sedative, anti‐inflammatory, antimicrobial, and antiviral activities.^[^ ^9^, ^10^ ^]^ However, the utilization of cannabis flowers and leaves and their extracts is preferred by medicinal users over the utilization of single phytocannabinoids.^[^ ^11^ ^]^ Concerning this, the biopotency of cannabis extracts is associated with a distinct combination of phytocannabinoid, terpene, and phenolic constituents due to their synergistic and/or entourage interactions.^[^ ^11^, ^12^ ^]^

In humans, Δ^9^‐THC and CBD bind to G protein‐coupled phytocannabinoid receptors CB1 and CB2 on cell membranes and modulate second messengers and signaling components such as adenylate cyclase, mitogen‐activated protein kinases (MAPK), and members of the nuclear factor κB (NF‐κB) family as well cell membrane ion channels.^[^ ^13^ ^]^ While their spasmolytic and sedative effects are mediated via CB1 receptors that are expressed predominantly in the brain and some peripheral tissues, their anti‐inflammatory effects are mediated via CB2 receptors that are predominantly expressed in immune cells.^[^ ^14^ ^]^

SARS‐CoV‐2 is a positive‐sense single‐stranded RNA‐enveloped virus consisting of a lipid bilayer and four structural proteins, the spike (S), membrane (M), envelope (E), and nucleocapsid (N) protein.^[^ ^2^ ^]^ These proteins promote virion budding and recruit the protein and the viral genomic RNA into nascent virions. S protein binds to the angiotensin‐converting enzyme 2 (ACE2) receptor, and upon proteolytic activation, its two noncovalently bound subunits S1 and S2 facilitate viral entry into the host cell.^[^ ^15^ ^]^ Among nonstructural proteins, SARS‐CoV‐2 main protease (MPro) plays a key role in viral replication along with the papain‐like protease, processing the translated viral polyproteins into functional proteins.^[^ ^15^ ^]^ To date, few compounds have been identified as the S protein and MPro inhibitors.^[^ ^16^ ^]^

CBD, Δ^9^‐THC and their corresponding carboxylic acids, Δ^9^‐tetrahydrocannabinolic acid (Δ^9^‐THCA), and cannabidiolic acid (CBDA) could preclude SARS‐CoV‐2 lifecycle in infected human cells via their interactions with S protein/ACE2 complexes and MPro.^[^ ^17^ ^]^ Different in silico and in vitro studies showed that these phytocannabinoids can bind to the orthosteric site of the S1 subunit of S protein at micromolar concentrations and thereby preclude its interaction with pre/ACE2.^[^ ^18^ ^]^ Specifically, only CBD inhibits viral replication by upregulating the host inositol‐requiring enzyme‐1α (IRE1α) mediated endoplasmic reticulum (ER) stress response and interferon signaling pathways.^[^ ^19^ ^]^ Most significantly, both in silico and in vitro studies showed that CBD, Δ^9^‐THC, Δ^9^‐THCA, and CBDA can inhibit MPro activity and viral replication.^[^ ^20^ ^]^ These four phytocannabinoids have been found to bind with a high affinity to the different sites of the active pocket of MPro via hydrogen bonds and π‐interactions, and four interacting amino acids residues GLN189, MET165, and GLU166 are required for this binding.^[^ ^20^ ^]^ However, CBD and Δ^9^‐THC are more effective than their corresponding carboxylic acids in inhibiting viral replication, and the values of IC_50_ against SARS‐CoV‐2 are comparable to those of reference drugs like chloroquine and remdesivir (IC_50_ ≤ 10 µM). In addition, the antiviral activity was also observed for phytocannabinoid formulations as well as for a cannabis extract.^[^ ^21^ ^]^

There is an increasing interest in the possibility of utilizing cannabis extracts in complementary antiviral treatments for COVID‐19.^[^ ^22^ ^]^ Various cannabis extracts differ in secondary metabolite profiles, and consequently, their antiviral property might vary.^[^ ^23^ ^]^ Therefore, in this study, the antiviral activities against SARS‐CoV‐2 and constituent profiles of cannabinoid extracts from seven high‐CBD and two high‐THC genotypes of *C. sativa* were assessed.

## 2 RESULTS

### 2.1 Cannabinoid extracts

The differences between the phytocannabinoid extracts of the CBD and THC *Cannabis* genotypes in phytoconstituent composition and antiviral activity against SARS‐CoV‐2 were determined in this study. For easier data interpretation, the seven acidic extracts of Elleta Campana, Harlequin, Strawberry, Mandarin, Lemon Heaven, Lemon Master, and Fantasy Bud high‐CBD *C. sativa* genotypes were named CBD1, CBD2, CBD3, CBD4, CBD5, CBD6, and CBD7, respectively, while the seven different neutral extracts prepared from decarboxylated plant material of the Elleta Campana, Harlequin, Strawberry, Mandarin, Lemon Heaven, Lemon Monster, Lemon Heaven, and Fantasy Bud were named CBD‐1D, CBD‐2, CBD3‐D, CBD4‐D, CBD5‐D, CBD6‐D and CBD7‐D, respectively. Likewise, the acidic extracts of Kosher Haze and Prima Holandica high‐THC *C. sativa* genotypes were named THC1 and THC2, respectively, while the neutral extracts of Kosher Haze and Prima Holandica were named THC1‐D and THC2‐D, respectively.

### 2.2 Chemical composition of cannabinoid extracts

A total of 12 phytocannabinoids were detected and quantified in the 18 investigated phytocannabinoid extracts by LC‐UV analysis (Supporting Information: Table S3). The major compound in the CBD extracts was CBDA, ranging from 6.80 ± 0.14 mg/mL to 14.40 ± 0.17 mg/mL for the CBD2 and CBD1, respectively. These extracts also contained minor amounts of Δ^9^‐THCA, CBCA, and CBD whose concentrations did not reach a concentration higher than 1 mg/mL of the extract, except the CBD concentration in CBD1 and CBD7 extracts. The CBD‐D extracts prepared from the decarboxylated inflorescences still contained certain amounts of CBDA, ranging from 0.98 ± 0.02 mg/mL to 3.10 ± 0.03 mg/mL in CBD2‐D and CBD7‐D, respectively. The CBD content in these neutral extracts increased by 10–20‐fold, and ranged from 5.95 ± 0.04 mg/mL (CBD2‐D) to 13.55 ± 0.25 mg/mL (CBD4‐D). The phytocannabinoid levels of THC and THC‐D extracts of two different high‐THC *C. sativa* genotypes, Kosher Haze (THC1 and THC1‐D) and Prima Holandica (THC2 and THC2‐D), were also significantly different. The concentrations of Δ^9^‐THCA were 15.19 ± 0.04 mg/mL and 16.82 ± 0.50 mg/mL in THC1 and THC2, respectively, while the concentrations of Δ^9^‐THC were only 1.30 ± 0.06 mg/mL and 2.32 ± 0.31 mg/mL in THC1 and THC2, respectively. However, the amounts of Δ^9^‐THCA in THC‐D extracts decreased to 1.17 ± 0.01 mg/mL and 0.55 ± 0.00 mg/mL in THC1‐D and THC2‐D, respectively, while the amount of Δ^9^‐THC increased to 14.04 ± 0.14 mg/mL and 17.71 ± 0.33 mg/mL in THC1‐D and THC2‐D, respectively, as a consequence of the decarboxylation. The presence of other phytocannabinoids in THC and THC‐D extracts was minor, especially in THC‐D ones.

The results of extensive LC‐MS/MS analyses showed distinct levels of phenolic compounds in the extracts that are summarized in Supporting Information: Tables S4 and S5. Among phenolic acids, salicylic acid (SaA) and its glucoside (SaAG) were the most abundant compounds in all extracts prepared from CBD genotypes. However, their levels were significantly lower in the extracts prepared from THC genotypes. The CBD7 extract contained the least SaA amount (61.22 ± 1.18 ng/mL) in contrast to the highest SaA concentration (367.59 ± 3.52 ng/mL) in the CBD2 extract, while SaAG concentration ranged from 32.38 ± 2.08 ng/mL to 618.78 ± 18.62 ng/mL, for the same extracts mentioned above. Generally, hydroxybenzoic acids were predominant to hydroxycinnamic acids in all extracts used in this study. The other abundant representatives of hydroxybenzoic acids were vanillic (VA) and 4‐hydroxybenzoic acid (4HBA) (Supporting Information: Table S4). The concentrations of all phenolic acids significantly decreased in CBD‐D extracts, which implies they are decomposed during the decarboxylation process. On the contrary, the most abundant acid in THC extracts was VA, whose concentration levels were similar to those in CBD extracts. Although levels of all phenolic acids in extracts from high‐THC *C. sativa* genotypes were notably lower than those of high‐CBD *C. sativa* genotypes, there was no significant change in their concentrations when high‐THC plant material was subjected to decarboxylation (Supporting Information: Table S4).

The investigated phytocannabinoid extracts were rich in flavonoids, with orientin (ORI), luteolin (LUT), and cannflavin A (CANN A) as the main representatives (Supporting Information: Table S5). The LC‐MS/MS analyses showed that their levels were significantly higher than the levels of phenolic acids. Again, acidic extracts obtained from high‐THC *C. sativa* genotypes had lower levels of these phytoconstituents. Remarkably, a significant decrease in their content was observed following the decarboxylation process of high‐CBD *C. sativa* genotypes, particularly noticeable for rutin (RUT), vitexin (VIT), LUT, and both cannflavins A and B. Conversely, orientin (ORI) levels rose after the heat treatment, indicating potential liberation from its bound forms with other plant constituents or conversion from other compounds. However, it is reasonable to assume that overall, a substantial loss of phenolic compounds occurred during heating. Despite the possibility of heat‐induced release of free compounds from their bound forms with plant constituents, phenolic compounds are believed to be thermally unstable, and high temperatures may lead to their decomposition.^[^ ^24^ ^]^

Regarding terpene composition (Supporting Information: Table S6), between 83.8 ± 0.3% and 92.4 ± 0.8% of the total chromatographic peak area corresponding to the terpenes is identified successfully by GC‐MS analyses in the studied extracts using the library database.^[^ ^25^ ^]^ The main volatile compounds in CBD extracts were sesquiterpenes β‐caryophyllene (from 4.8 ± 0.0% to 18.0 ± 0.2%), 10‐*epi*‐γ‐eudesmol (from 7.9 ± 0.1% to 12.2 ± 0.1%), guaiol (from 7.3 ± 0.0% to 15.0 ± 0.0%), and α‐bisabolol (from 1.3 ± 0.0% to 18.1 ± 0.1%). THC extracts slightly differed in terpenoid profiles from CBD extracts, mainly due to the absence of guaiol, but also significantly lower levels of α‐bisabolol, and, conversely, notably higher levels of selina‐4(15),7(11)‐diene, eudesma‐5,7(11)‐diene, and selina‐3,7(11)‐diene. Among monoterpenes, significant levels of myrcene and linalool were observed (up to 5.8 ± 0.1% and 6.9 ± 0.1%, respectively), but still lower than the abovementioned sesquiterpenes. Extracts from high‐THC *C. sativa* genotypes displayed lower percentages of identified areas, primarily due to the presence of unknown sesquiterpenes in greater abundance. Changes in terpene composition following the decarboxylation of extracts from high‐CBD *C. sativa* genotype extract were observed. Consequently, a significant reduction in monoterpene content after heating the plant material, notably α‐pinene, β‐pinene, myrcene, and limonene, was detected. This decrease in monoterpene content was reflected in the higher abundance of sesquiterpenes in the chemical profile.

**Figure 1:**
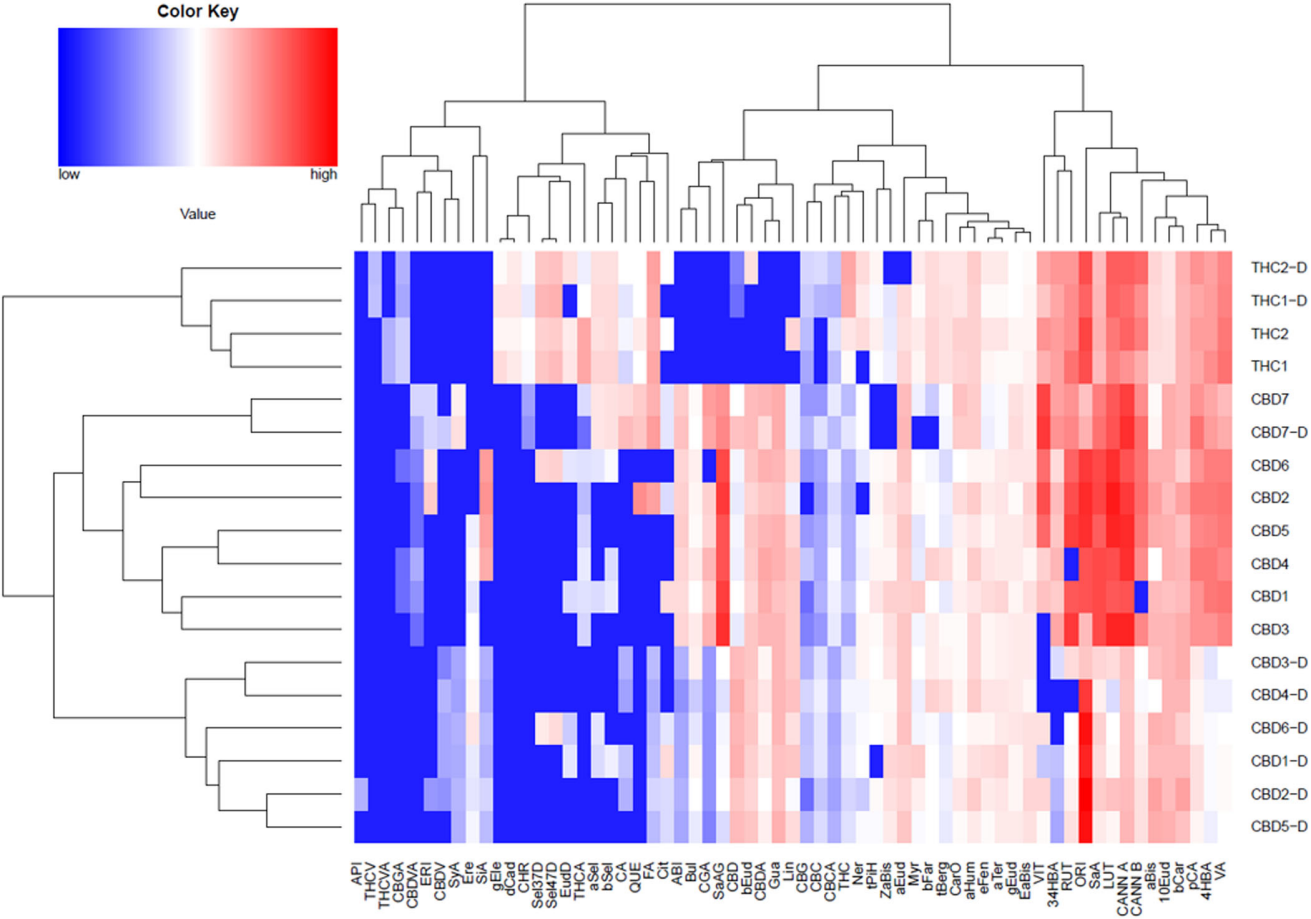
Heatmap of the phytocannabinoid, phenolic, and terpene levels in investigated *Cannabis* extracts. The seven acidic extracts of Elleta Campana, Harlequin, Strawberry, Mandarin, Lemon Heaven, Lemon Master, and Fantasy Bud high‐CBD *C. sativa* genotypes were named CBD1, CBD2, CBD3, CBD4, CBD5, CBD6, and CBD7, respectively. The acidic extracts of Kosher Haze and Prima Holandica high‐THC *C. sativa* genotypes were named THC1 and THC2, respectively. Decarboxylated extracts were prepared from decarboxylated inflorescences. They were labeled with their corresponding aforementioned extract names and suffix ‐D. Compounds: cannabidiolic acid (CBDA), Δ^9^‐tetrahydrocannabinolic acid (THCA), cannabidivarinic acid (CBDVA), tetrahydrocannabivarinic acid (THCVA), cannabigerolic acid (CBGA), cannabichromenic acid (CBCA), cannabidiol (CBD), Δ^9^‐tetrahydrocannabinol (THC), cannabigerol (CBG), cannabichromene (CBC), cannabidivarin (CBDV), tetrahydrocannabivarin (THCV), 3,4‐dihydroxybenzoic acid (34DHBA), salicylic acid glucoside (SaAG), chlorogenic acid (CGA), vanillic acid (VA), caffeic acid (CA), syringic acid (SyA), 4‐hydroxybenzoic acid (4HBA), *p*‐coumaric acid (pCA), salicylic acid (SaA), ferulic acid (FA), sinapic acid (SiA), orientin (ORI), vitexin (VIT), abietin (ABI), rutin (RUT), eriodyctiol (ERI), luteolin (LUT), apigenin (API), cannflavin B (CANN B), cannflavin A (CANN A), quercetin (QUE), chrysoeriol (CHR), myrcene (Myr), limonene (Lim), fenchone (FEN), linalool (Lin), *endo*‐fenchol (eFen), *trans*‐pinene hydrate (tPiH), ipsdienol (Ips), borneol (Bor), α‐terpineol (aTer), hexyl butanoate (HBut), citronellol (Cit), hexyl hexanoate (HHex), β‐caryophyllene (bCar), γ‐elemene (gEle), α‐*trans*‐bergamotene (tBerg), α‐humulene (aHum), (*E*)‐β‐farnesene (bFar), γ‐muurolene (gMur), β‐selinene (bSel), α‐selinene (aSel), (*Z*)‐α‐bisabolene (ZaBis), β‐curcumene (bCur), δ‐cadinene (dCad), β‐sesquiphellandrene (bSes), selina‐4(15),7(11)‐diene (Sel47D), eudesma‐5,7(11)‐diene (EudD), selina‐3,7(11)‐diene (Sel37D), (*E*)‐α‐bisabolene (EaBis), germacrene B (Ger), (*E*)‐nerolidol (Ner), caryophyllene oxide (CarO), guaiol (Gua), 5‐*epi*‐7‐*epi*‐α‐eudesmol (57Eud), 10‐*epi*‐γ‐eudesmol (10Eud), eremoligenol (Ere), γ‐eudesmol (gEud), amorph‐4‐en‐10α‐ol (Amo), β‐eudesmol (bEud), α‐eudesmol (aEud) 7‐*epi*‐α‐eudesmol (7aEud), bulnesol (Bul), α‐bisabolol (aBis), 5‐*neo*‐cedranol (nCed), (2*E*,6*Z*)‐farnesol (Farn).

To get a clearer picture of similarities and differences in the phytochemical composition of investigated extracts, both concentrations and the percentage content of all detected analytes were normalized using binary logarithm, and heatmap and principal component analysis (PCA) biplots were constructed using R‐studio software. Due to the complexity of the data, besides phytocannabinoids and phenolic compounds, terpenes in levels higher than 2% found in any of the samples were considered in this analysis. Both heatmap and PCA biplots explicitly separate CBD genotypes from THC genotypes. Interestingly, the separation of THC and CBD *C. sativa* genotypes in the heatmap (Figure 1) was not mainly supported by different levels of phytocannabinoids CBD(A) and Δ^9^‐THC(A) but also by selected phenolic compounds (flavonoids ORI, LUT, CANN A, CANN B, and RUT) and some terpenes (10‐*epi*‐γ‐eudesmol, α‐bisabolol, α‐ and β‐selinene). Six neutral extracts of CBD genotypes (CBD1‐D to CBD6‐D) are subclustered in one group, while genotype Fantasy Bud (CBD7‐D) is grouped with acidic forms of CBD extracts. This is mainly because of lower levels of phenolic compounds in this genotype, which were not significantly changed during the decarboxylation process.

**Figure 2:**
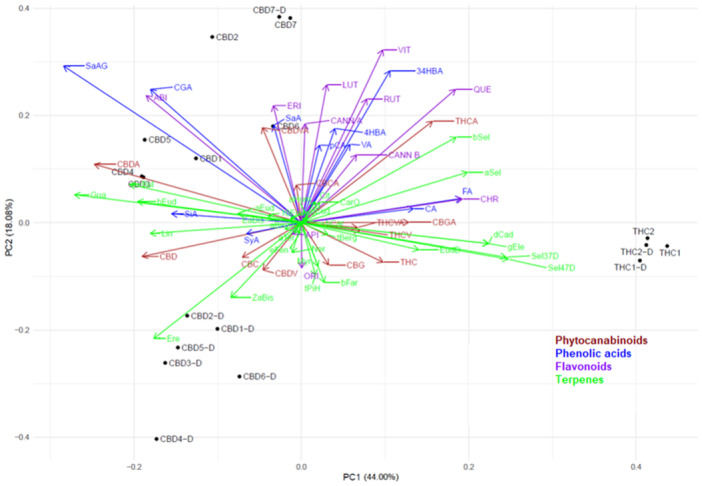
PCA biplot of the phytocannabinoid, phenolic, and terpene levels in investigated *Cannabis* extracts. The seven acidic extracts of Elleta Campana, Harlequin, Strawberry, Mandarin, Lemon Heaven, Lemon Master, and Fantasy Bud high‐CBD *C. sativa* genotypes were named CBD1, CBD2, CBD3, CBD4, CBD5, CBD6, and CBD7, respectively. The acidic extracts of Kosher Haze and Prima Holandica high‐THC *C. sativa* genotypes were named THC1 and THC2, respectively. Decarboxylated extracts were prepared from decarboxylated inflorescences. They were labeled with the corresponding aforementioned extract names and suffix ‐D. Compounds: cannabidiolic acid (CBDA), Δ^9^‐tetrahydrocannabinolic acid (THCA), cannabidivarinic acid (CBDVA), tetrahydrocannabivarinic acid (THCVA), cannabigerolic acid (CBGA), cannabichromenic acid (CBCA), cannabidiol (CBD), Δ^9^‐tetrahydrocannabinol (THC), cannabigerol (CBG), cannabichromene (CBC), cannabidivarin (CBDV), tetrahydrocannabivarin (THCV), 3,4‐dihydroxybenzoic acid (34DHBA), salicylic acid glucoside (SaAG), chlorogenic acid (CGA), vanillic acid (VA), caffeic acid (CA), syringic acid (SyA), 4‐hydroxybenzoic acid (4HBA), *p*‐coumaric acid (pCA), salicylic acid (SaA), ferulic acid (FA), sinapic acid (SiA), orientin (ORI), vitexin (VIT), abietin (ABI), rutin (RUT), eriodyctiol (ERI), luteolin (LUT), apigenin (API), cannflavin B (CANN B), cannflavin A (CANN A), quercetin (QUE), chrysoeriol (CHR), myrcene (Myr), limonene (Lim), fenchone (FEN), linalool (Lin), endo‐fenchol (eFen), *trans*‐pinene hydrate tPiH), ipsdienol (Ips), borneol (Bor), α‐terpineol (aTer), hexyl butanoate (HBut), citronellol (Cit), hexyl hexanoate (HHex), β‐caryophyllene (bCar), γ‐elemene (gEle), α‐*trans*‐bergamotene (tBerg), α‐humulene (aHum), (*E*)‐β‐farnesene (bFar), γ‐muurolene (gMur), β‐selinene (bSel), α‐selinene (aSel), Z)‐α‐bisabolene (ZaBis), β‐curcumene (bCur), δ‐cadinene (dCad), β‐sesquiphellandrene (bSes), selina‐4(15),7(11)‐diene (Sel47D), eudesma‐5,7(11)‐diene (EudD), selina‐3,7(11)‐diene (Sel37D), (*E*)‐α‐bisabolene (EaBis), germacrene B (Ger), (*E*)‐nerolidol (Ner), caryophyllene oxide (CarO), guaiol (Gua), 5‐*epi*‐7‐*epi*‐α‐eudesmol (57Eud), 10‐*epi*‐γ‐eudesmol (10Eud), eremoligenol (Ere), γ‐eudesmol (gEud), amorph‐4‐en‐10α‐ol (Amo), β‐eudesmol (bEud), α‐eudesmol (aEud) 7‐*epi*‐α‐eudesmol (7aEud), bulnesol (Bul), α‐bisabolol (aBis), 5‐*neo*‐cedranol (nCed), (2*E*,6*Z*)‐farnesol (Farn).

The PCA biplot (Figure 2) visualizes the relationships among diverse chemical compounds present in extracts, offering insights into the chemical profiles of selected *Cannabis* genotypes. The first two principal components represent 62.08% of the total data variance. Along the PC1 axis, phytocannabinoids exhibit noticeable dispersion, indicating substantial diversity in their contributions to the overall variance. CBD and CBDA appear on the left side of the plot, while Δ^9^‐THC and Δ^9^‐THCA are situated on the right, suggesting a negative correlation between them.

Phenolic acids predominantly imply a positive correlation between themselves and the majority of flavonoids but a negative correlation with certain phytocannabinoids, mainly Δ^9^‐THC, CBG, CBD, and CBDV. However, apigenin (API) and orientin (ORI) positively correlate with the abovementioned phytocannabinoids. In general, terpenes are also dispersed over the entire biplot, demonstrating significant variety in their impacts on the total variation. Therefore, comprehensive chemical analysis separates high‐THC *C. sativa* from high‐CBD *C. sativa* genotypes. Both acidic and neutral extracts of THC genotypes are closely grouped, indicating there are no significant changes in their composition during the decarboxylation process (except for Δ^9^‐THCA/Δ^9^‐THC conversion). This is not the case for extracts from high‐CBD *C. sativa* genotypes. Extracts prepared from decarboxylated plant material are grouped and separated from those prepared from original plant material. The only exception is the Fantasy Bud *C. sativa* genotype, whose composition also did not significantly change during decarboxylation (again, except for CBDA/CBD conversion). Also, it is important to emphasize that acidic CBD extracts showed higher variability between themselves than neutral CBD‐D extracts.

### 2.3 Cytotoxic activity of cannabinoid extracts

All of the acidic and neutral extracts of seven different CBD genotypes and two different THC *Cannabis* genotypes, that is, Elleta Campana, Harlequin, Strawberry, Mandarin, Lemon Heaven, Lemon Master, and Fantasy Bud, and Kosher Haze and Prima Holandica, had relatively small cytotoxicity to Vero 6 cells as the C_50_ values of their most abundant phytocannabinoids and corresponding reference control phytocannabinoids, CBD, CBDA, Δ^9^‐THC, and Δ^9^‐THCA were bigger than the 25 µM concentration (Supporting Information: Figure S1, Supporting Information: Table S7).

### 2.4 Antiviral activity of cannabinoid extracts

**Figure 3:**
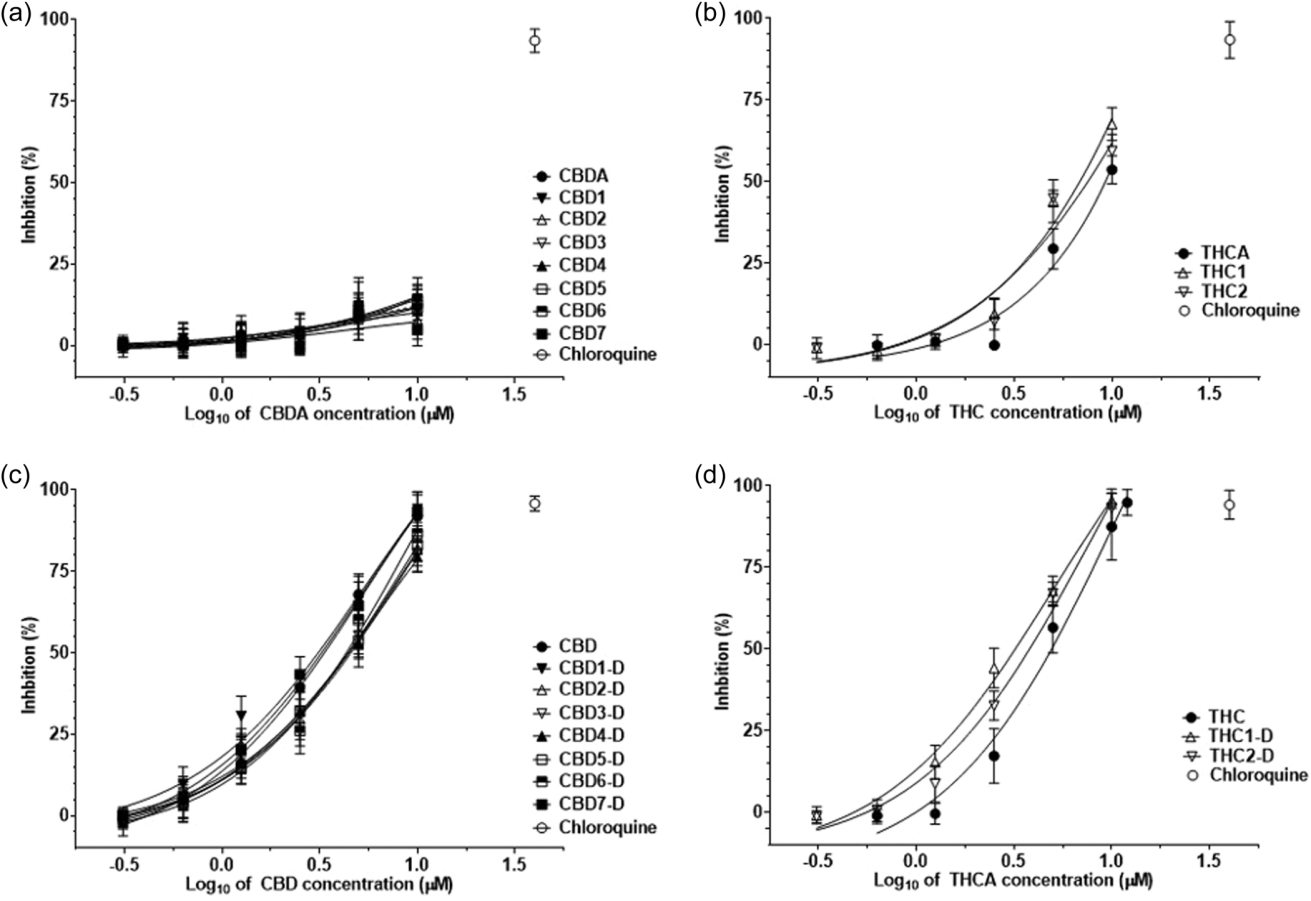
Antiviral activity of *Cannabis* extracts and single phytocannabinoids against SARS‐CoV‐2. The seven acidic extracts of Elleta Campana, Harlequin, Strawberry, Mandarin, Lemon Heaven, Lemon Master, and Fantasy Bud high‐CBD *C. sativa* genotypes were named CBD1, CBD2, CBD3, CBD4, CBD5, CBD6, and CBD7, respectively (a). The acidic extracts of Kosher Haze and Prima Holandica high‐THC *C. sativa* genotypes were named THC1 and THC2, respectively (b). Decarboxylated extracts were prepared from decarboxylated inflorescences (c,d). They were labeled with the corresponding aforementioned extract name and suffix ‐D. The data points represent the means of three biological replicates with three technical replicates. 30 μM chloroquine was used as a reference control antiviral drug.

A marked difference in antiviral activity against SARS‐CoV‐2 was found between acidic extracts of high‐CBD and high‐THC *C. sativa* genotypes, namely CBD and THC extracts. The acidic CBD extracts of seven different high‐CBD *C. sativa* genotypes, namely Elleta Campana, Harlequin, Strawberry, Mandarin, Lemon Heaven, Lemon Master, and Fantasy Bud, and the reference standard CBDA were inactive against SARS‐CoV‐2 as they did not inhibit the viral replication in the infected Vero 6 cells (Figure 3a), while the THC extracts of two high‐THC *C. sativa* genotypes, Kosher Haze (THC1) and Prima Holandica (THC1), and the reference standard of Δ^9^‐THCA had limited antiviral activity, and only at the 50 µM concentration, they inhibited the SARS‐CoV‐2 at the rate of 67.72 ± 3.42%, 59.97 ± 6.73%, and 53.27 ± 3.73%, respectively (Figure 3b). On the other hand, all neutral extracts of the seven different CBD and two different high‐THC *C. sativa* genotypes, as well as the reference standards of CBD and Δ^9^‐THC, exhibited formidable activity against SARS‐CoV‐2. They inhibited the viral replication in a dose‐dependent manner, and the IC_50_ values of their corresponding most abundant phytocannabinoids were smaller than the 10 µM concentration corresponding to those of the reference antiviral compounds like chloroquine (Table 1, Figure 3c,d). Thus, the obvious differences in the antiviral activity of the decarboxylated and non‐decarboxylated plant extracts showed the profoundly significant effect of decarboxylation on their antiviral property against SARS‐CoV‐2.

**Table 1:** The antiviral activity of *Cannabis* extracts and single cannabinoids.

Extract	Genotype	Sample code	IC_50_ (µM)	Student *t* test *p* values
Acid form	Elleta Campana	CBD1	NA	NA
	Harlequin	CBD2	NA	NA
	Strawberry	CBD3	NA	NA
	Mandarin	CBD4	NA	NA
	Lemon Heaven	CBD5	NA	NA
	Lemon Master	CBD6	NA	NA
	Fantasy Bud	CBD7	NA	NA
	Kosher Haze	THC1	LA	NA
	Prima Holandica	THC2	LA	NA
Neutral form	Elleta Campana	CBD1‐D	5.42 ± 1.03	0.6625
	Harlequin	CBD2‐D	6.11 ± 0.64	0.1657
	Strawberry	CBD3‐D	8.86 ± 0.20	<0.0071**
	Mandarin	CBD4‐D	6.41 ± 0.51	0.0823
	Lemon Heaven	CBD5‐D	7.92 ± 0.97	0.0191
	Lemon Master	CBD6‐D	8.75 ± 0.46	<0.0071**
	Fantasy Bud	CBD7‐D	5.19 ± 0.29	0.8079
	Kosher Haze	THC1‐D	5.22 ± 0.56	<0.025*
	Prima Holandica	THC2‐D	8.38 ± 1.47	0.1405
Standard control		CBD	5.05 ± 0.88	
		THC	10.71 ± 1.64	
		CBDA	NA	
		THCA	LA	

NA, inactive compound in a tested range of a serial of twofold 10.0–0.31 μM dilutions of their most abundant cannabinoid compound; LA, compound with limited activity with the inhibition rate above 50%; *p* values from Student *t* test which refer to comparison with reference standard control.

The average IC_50_ values against SARS‐CoV‐2 of the neutral extracts of Elleta Campana, Harlequin, Mandarin, Lemon Heaven, and Fantasy Bud were not significantly different from that of the reference standard CBD control (unpaired Student *t* test: in all cases, *p* > 0.0071; Table 1). In contrast, the average IC_50_ values of the neutral extracts of Strawberry and Lemon Master genotypes (CBD3‐D and CBD6‐D extracts, respectively) were significantly higher than that of the CBD reference standard (unpaired Student *t* test: *p* < 0.007; Table 1), showing their decreased activity. Such decreased activity was also close to the point of significance in the neutral extract of Lemon Heaven, CDB5‐D extract (unpaired Student *t* test: *p* = 0.0191). On the other hand, comparing the average IC_50_ values against SARS‐CoV‐2 of neutral extracts of the Kosher Haze (THC1‐D) and Prima Holandica (THC2‐D) genotypes with that of the reference standard Δ^9^‐THC were 2.05 and 1.23 times smaller than that of the reference standard Δ^9^‐THC though only the value for THC1‐D extract was significantly different (unpaired Student *t* test: *p* < 0.025; Table 1). Such a gradual increase in antiviral activity of these two THC‐D extracts correlates with differences in the level of other phytocannabinoids indicating additive interaction. Thus, while previous studies showed that the chemical composition of phytocannabinoid extracts varies among different *C. sativa* genotypes and can be modulated by decarboxylation, our data shows that their antiviral properties also depend on the genotype and sample preparation process including heating.

### 2.5 Correlation between bioactivity and phytoconstituents

**Figure 4:**
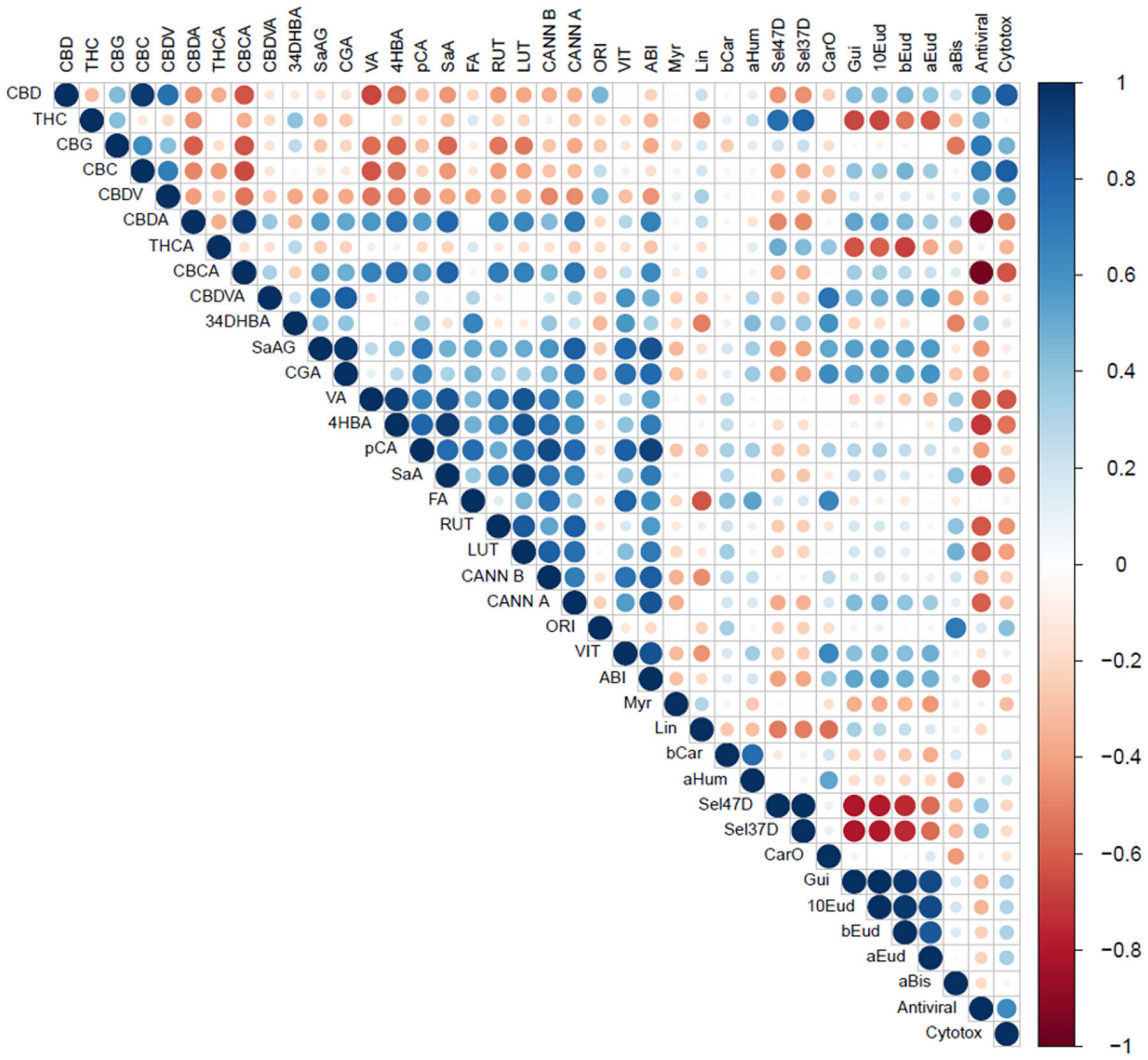
Correlation matrix between selected constituents of *Cannabis* extracts and their antiviral and cytotoxic activities. The selected constituents: cannabidiolic acid (CBDA), Δ^9^‐tetrahydrocannabinolic acid (THCA), cannabidivarinic acid (CBDVA), tetrahydrocannabivarinic acid (THCVA), cannabichromenic acid (CBCA), cannabidiol (CBD), Δ^9^‐tetrahydrocannabinol (THC), cannabigerol (CBG), cannabichromene (CBC), cannabidivarin (CBDV), cannabidivarinic acid (CBDVA), tetrahydrocannabivarin (THCV), 3,4‐dihydroxybenzoic acid (34DHBA), salicylic acid glucoside (SaAG), chlorogenic acid (CGA), vanillic acid (VA), 4‐hydroxybenzoic acid (4HBA), *p*‐coumaric acid (pCA), salicylic acid (SaA), ferulic acid (FA), orientin (ORI), vitexin (VIT), abietin (ABI), rutin (RUT), luteolin (LUT), cannflavin B (CANN B), cannflavin A (CANN A), myrcene (Myr), limonene (Lim), β‐caryophyllene (bCar), α‐*trans*‐α‐humulene (aHum), selina‐4(15),7(11)‐diene (Sel47D), selina‐3,7(11)‐diene (Sel37D), caryophyllene oxide (CarO), guaiol (Gua), 10‐*epi*‐γ‐eudesmol (10Eud), β‐eudesmol (bEud), α‐eudesmol (aEud), α‐bisabolol (aBis).

To assess which compounds in the extracts contributed to their antiviral activity, a Pearson correlation matrix between the antiviral and cytotoxic activities and the chemical composition of the investigated extracts was created (Figure 4). For these correlations, the exact amount of each component for each particular extract (Supporting Information: Tables S5 and S6) was used, as well as their inhibitory effects on SARS‐CoV‐2 and cytotoxic effects on Vero 6 cells (Figure 4). Pearson's correlation analyses showed that six phytocannabinoids, particularly CBD, CBC, Δ^9^‐THCA, Δ^9^‐THC, CBG, and CBDV, can be associated with the antiviral activity of the tested extracts (Figure 4). However, this finding should be interpreted cautiously. In the THC1‐D extract, three phytocannabinoids (Δ^9^‐THCA, CBG, and CBC) might contribute to the additive interactions with the main phytocannabinoid. In contrast, the antiviral activity in the neutral extracts of all other *C. sativa* genotypes is due solely to their main phytocannabinoid (Figures 2, 3, 4). Interestingly, Pearson's correlations analyses showed that among all identified nonphytocannabinoid compounds, three phenolic acids, salicylic acid (SaA) and its glucoside SaAG, chlorogenic acid (CGA), and ferulic acid (FA), and two flavonoids, abietin (ABI) and luteolin (LUT), are implicated in the antagonistic effects on predominant phytocannabinoids of the investigated neutral extracts of the CBD genotypes (Table 1, Figure 4). In addition, in the present study, selected phytocannabinoid acids, namely CBDA (cannabidiolic acid) and CBCA (cannabichromenic acid), showed a strong negative correlation with the antiviral activity (Figure 4). The flavonoid orientin (ORI) that is abundant in the investigated extracts was also associated with antiviral activity (Figure 4). However, Pearson's correlation analyses suggest that ORI may also cause cytotoxicity to Vero‐6 cells, so its contribution to the antiviral properties of the investigated extracts cannot be confirmed (Figure 4). Among all, two identified sesquiterpenes, namely β‐eudesmol (bEud) and α‐humulene (aHum), correlate with the cytotoxicity of the tested phytocannabinoid extracts (Figures 2 and 4). However, their levels as well as those of other terpenes in the investigated plant extracts were too low to exhibit any bioactivity.

## 3 DISCUSSION

Chemical profiling and bioactivity assessment of cannabinoid extracts can lead to the development of cannabinoid extract formulations that could be used for the treatment of different medical conditions and diseases, including different infectious diseases.^[^ ^26^, ^27^ ^]^ Therefore, in our study, we determined the three secondary metabolite profiles, cannabinoid, terpenoid, and phenolic profiles, and antiviral activity against SARS‐CoV‐2 of cannabinoid extracts from seven different CBD and two THC *C. sativa* genotypes. Most importantly, our results show that the acidic extracts of two high‐THC genotypes and the neutral phytocannabinoid extracts of all seven CBD and two high‐THC genotypes inhibited the SARS‐CoV‐2 in the infected Vero cells (Figure 3, Table 1). Thus, these cannabinoid extracts have the antiviral property that is required for the development of advanced cannabinoid formulations.

The decarboxylation caused a significant conversion in the phytocannabinoid profiles of the extracts, and the neutral forms, CBD and Δ^9^‐THC, became their predominant phytocannabinoids (Supporting Information: Table S3). Such conversion in phytocannabinoid profiles is directly associated with the enhancement of the antiviral activity (Figure 3, Table 1). This can be also corroborated with the results of the previous studies of Raj et al.^[^ ^20^ ^]^ that reported the antiviral activity of CBD and Δ^9^‐THC, with IC_50_ values of 7.91 and 10.25 µM, respectively.

While the antiviral activity of the neutral phytocannabinoid extracts is attributable to their single predominant cannabinoids, their non‐phytocannabinoid components, phenolic compounds, flavonoids, and terpenoids, might be implicated in pharmacological profiles to alleviate different facets of COVID‐19. Surprisingly, there are numerous publications dealing with cannabinoid profiles of different *C. sativa* genotypes,^[^ ^28^, ^29^ ^]^ but there are just a few recent studies on the other bioactive phytoconstituent profiles such as phenolic compounds and terpenoid profiles.^[^ ^30^, ^31^, ^32^, ^33^ ^]^ Specifically, CBD extracts showed high levels of flavonoids including orientin, luteolin, and cannflavins (Supporting Information: Table S5). On the other hand, all the investigated extracts had a diverse array of sesquiterpenes. Among them, the most abundant were β‐caryophyllene, 10‐*epi*‐γ‐eudesmol, and α‐bisabolol (Supporting Information: Table S6). Subsequently, these results can also be corroborated with those of previous studies that showed that all of the aforementioned flavonoids and sesquiterpenes have proven anti‐inflammatory, antioxidant, antiviral, and antimicrobial properties.^[^ ^34^, ^35^ ^]^ A recent review by Jha et al.^[^ ^36^ ^]^ emphasizes that β‐caryophyllene exhibits antiviral and anti‐inflammatory properties, potentially beneficial in COVID‐19 treatment. However, in our study, this sesquiterpene did not show a correlation with the antiviral activity of the examined extracts.

Our finding that the antiviral activity against SARS‐CoV‐2 of the single predominant phytocannabinoids of the neutral extracts of the high‐CBD and high‐THC *C. sativa* is modulated by other extract components shows the presence of the pharmacodynamic interactions among components of these phytocannabinoid extracts (Table 1), specifically. Furthermore, principal component analyses indicated that five cannabinoids, CBD, Δ^9^‐THC, CBG, CBC, and CBDV, could be implicated in additive interactions (Figure 4). These findings can be also corroborated by the findings of the previous studies that showed while the isolated or synthetic phytocannabinoids can exhibit biological activity, their biopotency and therapeutic effect alone are not so great as those of cannabis extracts or flowers with complex chemical compositions where the synergistic and additive interactions can occur.^[^ ^11^, ^12^ ^]^

Specifically, concerning phytocannabinoid extracts, two types of “entourage effects” have been defined with their components: “intra‐entourage” and “inter‐entourage.”^[^ ^37^ ^]^ In its initial definitions, the former refers only to phytocannabinoid‐to‐phytocannabinoid or terpene‐to‐terpene interactions, while the latter refers to phytocannabinoid‐to‐terpene interactions.^[^ ^11^, ^12^ ^]^ In our study, the antiviral activity of the investigated extracts is generally due to single phytocannabinoids. Therefore, the intra‐entourage effects cannot be linked to their antiviral activity. The potential inter‐entourage effect between phytocannabinoids and terpenes in different bioactivities is thoroughly discussed in the review of Russo.^[^ ^11^ ^]^ Due to significant differences in the levels of cannabinoids and terpenes in the extracts, the contribution of the latter to the antiviral activity is questionable. Previous studies also showed that terpenes and phenolic compounds including flavonoids possess cannabimimetic properties and may synergistically interact with phytocannabinoids as CB1 and CB2 receptor agonists.^[^ ^38^, ^39^ ^]^ However, such synergistic effects between different phenolic acids, flavonoids, and phytocannabinoids are not involved in the antiviral activity against SARS‐CoV‐2 of the investigated phytocannabinoid extracts. In our study, we investigated the antiviral properties of 18 different phytocannabinoid extracts exclusively against the SARS‐CoV‐2 virus while the activity of these phytocannabinoid extracts might differ against the other viruses. Future studies on the activity of the phytocannabinoid extracts against other viruses might show whether the intra‐entourage and the extra‐entourage effects occur in their antiviral properties.

The finding that three phenolic acids, salicylic acid (SaA) and its glucoside (SaAG), chlorogenic acid (CGA), and ferulic acid (FA), and two flavonoids, abietin (ABI) and luteolin (LUT), are implicated in the antagonistic effects on the single predominant phytocannabinoid of some neutral extracts of the CBD genotypes (Table 1, Figure 4) can be corroborated by the results of previous studies that these acidic compounds can interfere with the absorption and availability of phytocannabinoids in the treated cells.^[^ ^40^ ^]^ Furthermore, its relevance is highlighted by the fact that the content of these phenolic acids in these neutral extracts varies with genetic differences among CBD (Table 1).

More recently, the metabolic study of Wishart et al.^[^ ^41^ ^]^ showed that phenolic compositions of *C. sativa* are also implicated in the pharmacological profiles of phytocannabinoid extracts and created the interactive cannabis component database. This database gives comprehensive insights into the chemical composition of cannabis, offering valuable data for both scientific and cannabis communities. Therefore, our results on the phytoconstituent composition and antiviral properties of different *C. sativa* genotypes can contribute to the biochemical characterization of *Cannabis* genotypes and help in their chemo‐taxonomical classification.

## 4 CONCLUSION

The presented study contributes to the knowledge of the medicinal properties of *C. sativa*, and it implies that phytocannabinoid extracts could also be used in supplementary treatments for COVID‐19. The findings show that the non‐decarboxylated phytocannabinoid extracts of two THC genotypes and all decarboxylated extracts have in vitro antiviral properties against SARS‐CoV‐2. Among these phytocannabinoid extracts, there are differences in the antiviral activities; the decarboxylated phytocannabinoid extracts have greater antiviral potency than the non‐decarboxylated extracts. Our results indicate that five cannabinoids, CBD, Δ^9^‐THC, CBG, CBC, and CBDV, could be implicated in the additive interactions to enhance the antiviral activity of predominant cannabinoids and that the phenolic acids might have antagonistic effects on the predominant cannabinoids. However, they also show that the phytocannabinoid extracts have flavonoid and terpenoid contents that could enrich their pharmacological profiles. Based on the differences in phytoconstituent profiles, the principal component analyses separated the CBD genotypes from the THC ones and revealed further separations within CBD genotypes. This emphasizes the significance of considering the genetic diversity in *C. sativa* strains when assessing their biomedical properties.

## 5 FUTURE PERSPECTIVES

Our studies showed that neutral extracts of different *C. sativa* genotypes have antiviral properties against SARS‐CoV‐2 though further preclinical studies on formulations of these extracts are needed to enhance their antiviral potential. One direction of these studies might be to obtain the extract formulations with modified non‐phytocannabinoid compositions and asses their antiviral potential.

## 6 EXPERIMENTAL

### 6.1 Plant material

The clones of CBD genotype ˝Fantasy Bud˝ were purchased from Konopex company. The high‐THC plants were propagated from the seeds (˝Prima Holandica,˝ ˝Kosher Haze˝; Dutch Passion®) and cultivated indoors at the Crop Research Institute in Olomouc. Other CBD genotypes (˝Eletta,˝ ˝Harlequin,˝ ˝Strawberry,˝ ˝Mandarin,˝ ˝Lemon Heaven,˝ ˝Lemon Master˝) were obtained from Farmer's Therapy, s.r.o.

### 6.2 Chemical reagents

Stock solutions of pure certified analytical standards (Cerilliant®) of cannabigerol (CBG), cannabigerolic acid (CBGA), cannabinol (CBN), cannabinolic acid (CBNA), ∆^8^‐tetrahydrocannabinol (∆^8^‐THC), cannabichromene (CBC), cannabichromenic acid (CBCA), ∆^9^‐tetrahydrocannabivarin (∆^9^‐THCV), ∆^9^‐tetrahydrocannabivarinic acid (∆^9^‐THCVA), cannabidivarin (CBDV), cannabidivarinic acid (CBDVA), cannabicyclol (CBL), and cannabicyclolic acid (CBLA) were purchased from Merck. Standards of (‐)‐*trans*‐∆^9^‐tetrahydrocannabinol ((‐)‐*trans*‐∆^9^‐THC), (‐)‐*trans*‐∆^9^‐tetrahydrocannabinolic acid ((‐)‐*trans*‐∆^9^‐THCA‐A), cannabidiol (CBD), and cannabidiolic acid (CBDA) were purchased from Lipomed.

Standard solutions of the phenolic compounds were first prepared in methanol at 1 mM concentrations, and solutions were gradually diluted in the mobile phase to the working concentrations that ranged from 0.01 to 50 µM. Each solution contained gallic acid, gallocatechin, 3,4‐dihydroxybenzoic acid, epigallocatechin, abietin, salicylic acid glucoside, chlorogenic acid, catechin, 4‐hydroxybenzoic acid, 2,3‐hydroxybenzoic acid, caffeic acid, vanillic acid, syringic acid, 3‐hydroxybenzoic acid, *p*‐coumaric acid, coniferyl alcohol, sinapyl alcohol, ferullic acid, salicylic acid, sinapic acid, isoorientin, orientin, myricitrin, vitexin, rutin, isovitexin, coniferyl aldehyde, sinapaldehyde, quercitrin, myricetin, rosmarinic acid, naringin, hesperidin, phloridzin, morin, eriodictyol, quercetin, *p*‐methyl coumarate, *trans*‐cinnamic acid, apigenin, naringenin, phloretin, luteolin, kaempherol, chrysoeriol, chrysin, pinocembrin, galangin, cannflavin A, and cannflavin B. All standards were of the highest available purity and purchased from Sigma Aldrich Company, except for cannflavin A and cannflavin B, which were purchased from Toronto Research Chemicals.

Solvents were from the following manufacturers: formic acid, Supelco® LC‐MS grade water (Merck), HPLC grade acetonitrile (Fisher Chemicals), 96% ethanol (Lach:ner), and *n*‐hexane (Sigma Aldrich).

### 6.3 Decarboxylation

Dried cannabis inflorescences, stripped of leaves and larger stems, were homogenized in a mortar bowl with a pestle and heated in closed glass bottles in a hot‐air dryer at 121°C for 30 min according to the conditions for medical cannabis recommended by Landa et al.^[^ ^42^ ^]^

### 6.4 Cannabis extract preparation

Non‐ or decarboxylated cannabis material was mixed with 96% ethanol in a flask in a ratio of 1:10 (cannabis: ethanol, w/v). Then, the sample was sonicated for 30 min and split into 15 mL plastic centrifuge tubes followed by centrifugation for 15 min (10,000*g*, laboratory temperature). The supernatant was transferred into 2 mL Eppendorf tubes. Each tube precisely contained 1 mL of the ethanolic extract. Finally, the solvent was evaporated in the centrifugal evaporator (CentriVap Solvent System; Labconco) and the extracts were stored in the freezer before the analysis or antiviral testing.

### 6.5 Phytocannabinoid analyses

The phytocannabinoid analysis was performed via ultra‐high performance liquid chromatography coupled to a UV detector (UHPLC‐UV) using an UltiMate™ 3000 UHPLC system (Thermo Fisher Scientific) according to the previously reported methodology.^[^ ^23^ ^]^ Separation was done on a Waters Cortecs UPLC C18 (100 × 2.1 mm, 1.6 μm particle size) column (Waters Corp.) kept at 35°C. The mobile phases were water (A) and acetonitrile (B), both containing 0.05% (v/v) formic acid. The flow rate was 0.3 mL/min and the injection volume was 10 µL. A binary gradient started at 70% B, held for 6 min, and increased to 100% B for 4.5 min and held for 0.2 min. Then, a decrease in B to 70% for 0.3 min followed. Finally, the column was re‐equilibrated to the initial conditions for 1.5 min. The wavelength of 228 nm was used for the detection and the Xcalibur 1.2 software (Thermo Fisher Scientific) was used for the data processing. For quantification, calibration solutions of 17 phytocannabinoid standards were measured.

The extract samples were prepared for the analysis as follows: 1.8 mL of 96% ethanol was added to the extract in a 2 mL Eppendorf tube and samples were sonicated for 15 min at laboratory temperature. After 10 min centrifugation (21,200*g*, laboratory temperature), the supernatant was diluted with 70% acetonitrile (ACN) containing 0.1% formic acid and filtrated over CHS FilterPure filters (Nylon filters, a diameter of 13 mm, the porosity of 0.22 μm; Chromservis). The quantified phytocannabinoids were expressed as the analyte amount in the extract (mg/mL).

### 6.6 Phenolic analyses

The chromatographic separation was performed on a BEH C18 reversed‐phase column (Acquity UPLC BEH C18, 2.1 × 100 mm, 1.7 μm) (Waters) maintained at 40°C. The flow rate was set to 0.4 mL/min and the injection volume was 5 μL. The mobile phase comprised 15 mM formic acid in water (pH 3) (A) and acetonitrile (B). The binary gradient consisted of 10% B for 1 min, 10%–13% B for 2 min, 13% B for 4 min, 13%–25% B for 3 min, 25%–70% B for 1.2 min, 70% B for 0.8 min, back to 10% B within 1.5 min, and re‐equilibration for 4 min. The electrospray source parameters were as follows: interface temperature of 300°C, heat block temperature of 400°C, and capillary voltage of 3.0 kV. Argon was used as the collision gas and nitrogen was used as the nebulizing gas. The analysis was performed on the Nexera X2 UHPLC instrument coupled to an MS‐8050 triple quadrupole MS (Shimadzu) and data were acquired and processed via the software LabSolutions v.5.97 SP1 (Shimadzu). The quantified phenolic compounds were expressed as amounts in the extract (ng/mL).

### 6.7 Terpenoid analyses

A 1.0 mL of 0.001% *n*‐tridecane (internal standard) in *n*‐hexane was added into the Eppendorf tube with the extract. The sample was sonicated for 30 min at room temperature and centrifuged for 10 min (21,200*g*, room temperature). Then, the supernatant was injected into a gas chromatography Agilent 7890A GC coupled with an HP 5975C MSD spectrometer (Agilent Technologies Inc.). Chromatographic separation was done on HP‐5MS UI (30.0 m × 250.0 µm; 0.25 µm film) (Agilent Technologies Inc.). The temperature program was as follows: from 60°C to 180°C, increasing 3°C/min (run time 40 min). Finally, the column was kept at 310°C for 10 min at post run. The flow rate of helium was 1.1 mL/min, and the injection volume was 1 µL. The temperatures of the injection and detector were 250°C and 230°C, respectively. Terpenoid identification was performed via comparison of the retention indices and mass spectra with literature data and Mass Finder 4.51 Computer Software as described in Adams et al.^[^ ^25^ ^]^ The terpene levels were expressed as their percentage composition in the extracts.

### 6.8 Cell and viral cultures

Vero 6 cells were obtained from the Czech Technical University in Prague. They were maintained in full Dulbecco growth medium (10% inactivated fetal calf serum (FCS), streptomycin (100 μg/mL), and penicillin (100 IU/mL) at 37°C at 5% CO_2_). The SARS‐CoV‐2 isolate from the first Czech patient was obtained from The National Institute of Public Health, Czechia.^[^ ^43^ ^]^

### 6.9 Cytotoxicity assay

The cytotoxicity of four single phytocannabinoids and phytocannabinoid extracts on Vero 6 cells and relevant IC_50_ values were determined by using an established MTS tetrazolium assay, as described in our previous study.^[^ ^44^ ^]^ All of the extracts were normalized by using the same amount of the most predominant phytocannabinoid in the final solutions, and a serial of extract dilutions was made to correspond to a 200.0–0.012 μM concentration range of a serial of fourfold dilutions of their most abundant phytocannabinoid compound (Supporting Information: Tables S1 and S2). Cytotoxicity was considered for the IC_50_ values below 50 μM. All single compounds were tested in a 25.0–0.012 μM concentration range of a series of fourfold dilutions.

### 6.10 Antiviral assay

The activity of four single cannabinoids and plant extracts against Vero 6 cells was determined by using a recently developed antiviral assay. All of the extracts were normalized by using the same amount of the most predominant cannabinoid in the final solutions, and a serial of compound extract dilutions was made to correspond to a 10.0–0.31 mM concentration of a serial of twofold dilutions of their most abundant cannabinoid compound (Supporting Information: Tables S6 and S7). The cells were incubated with compounds and extracts for 4 h before infection, then they were infected by SARS‐CoV‐2 at the multiplicity of infection (MOI) of 0.025 for 1 h. The viral inoculum was removed and the exact series of compound and extract dilutions that were used for pretreatment were placed into the wells for 96 h treatment. The MTT assay was used as described before.^[^ ^44^ ^]^

### 6.11 Statistical analyses

Experimental results were presented in tables and graphs as the mean ± standard deviation of three independent replications. Pearson correlations were performed to observe the correlation between the essential oil profile and cytotoxic and antiviral activity at the level of significance *p* < 0.05. PCA and correlation analysis were performed in RStudio (2023.12.0, Posit Software, PBC) using the following packages: *gplots*, *corrplot*, *RColorBrewer*, *ggrepel*, and *ggplot2*. The unpaired Student *t* test was performed using TIBCO Statistica (TIBCO Software Inc).

## CONFLICT OF INTEREST STATEMENT

The authors declare no conflict of interest.

## Supporting information

Supporting information.

## Data Availability

The data that support the findings of this study are available from the corresponding author upon reasonable request.
