# Supplementary material for: Secondary metabolite profiles and anti‐SARS‐CoV‐2 activity of ethanolic extracts from nine genotypes of Cannabis sativa L
Source: Arch Pharm (Weinheim). 2024 Nov 14;358(1):e2400607. doi: 10.1002/ardp.202400607 (PMC11726160; doi:10.1002/ardp.202400607)
Supplement: Supplementary file 1 — Supporting information. [file ARDP-358-e2400607-s001.docx]

**Supplemental Material**

Title: Secondary metabolite profiles and anti-SARS-CoV-2 activity of ethanolic extracts from nine genotypes of *Cannabis sativa* L.

Authors: Ermin Schadich^1≠*^, Dominika Kaczorová^2,3,4≠^, Tibor Béres^2^, Petr Džubák^1,2^, Marián Hajdúch^1,2^, Petr Tarkowski^2,3^, Sanja Ćavar Zeljković^2,3*^

1 Institute of Molecular and Translational Medicine, Faculty of Medicine and Dentistry, Palacký University, Olomouc, Czech Republic

2 Czech Advanced Technology and Research Institute (CATRIN), Palacký University, Olomouc, Czech Republic

3 Department of Genetic Resources for Vegetables, Medicinal and Special Plants, Crop Research Institute, Olomouc, Czech Republic

4 Department of Biochemistry, Faculty of Science, Palacký University, Olomouc, Czech Republic

*Correspondence:

Email: E-mail address

Dr, Ermin Schadich, Institute of Molecular and Translational Medicine, Faculty of Medicine and Dentistry, Palacký University, Hněvotínská 1333/5, 779 00 Olomouc, Czech Republic.

Email: ermin.schadich@upol,cz

Dr, Sanja Ćavar Zeljković, Czech Advanced Technology and Research Institute (CATRIN), Palacký University, Olomouc, Czech Republic

Email: sanja.cavar@upol.cz

**Statement on novel compounds**

There were no new compounds reported in thus study.

**Supplementary Table S1.** Dilutions of stock extract solutions for cytotoxic assays.

| **Extract** | **Genotype** | **Sample code** | **Stock solution**  **(mg/mL)** | **Dil. Factor** | ***Working solution (mg/mL)** | **Dil.**  **Factor** | **50.0**  **µM Final solution (µg/mL)** |
| --- | --- | --- | --- | --- | --- | --- | --- |
| Acid form | Elleta Campana | CBD1 | 14.4 | 4.017 | 3.585 | 803 | 1434.000 |
|  | Harlequin | CBD2 | 6.8 | 1.897 | 3.585 | 379 | 1434.000 |
|  | Strawberry | CBD3 | 9.75 | 2.72 | 3.585 | 544 | 1434.000 |
|  | Mandarin | CBD4 | 16.05 | 4.477 | 3.585 | 321 | 1434.000 |
|  | Lemon Heaven | CBD5 | 10.69 | 2.982 | 3.585 | 214 | 1434.000 |
|  | Lemon Master | CBD6 | 12.14 | 3.386 | 3.585 | 243 | 1434.000 |
|  | Fantasy Bud | CBD7 | 11.94 | 3.331 | 3.585 | 239 | 1434.000 |
|  | Kosher Haze | THC1 | 16.44 | 4.586 | 3.585 | 329 | 1434.000 |
|  | Prima Holandica | THC2 | 17.791 | 4.963 | 3.585 | 356 | 1434.000 |
| Neutral form | Elleta Campana | CBD1-D | 12.27 | 3.901 | 3.145 | 780 | 1258.000 |
|  | Harlequin | CBD2-D | 5.95 | 1.892 | 3.145 | 378 | 1258.000 |
|  | Strawberry | CBD3-D | 8.19 | 2.604 | 3.145 | 521 | 1258.000 |
|  | Mandarin | CBD4-D | 13.55 | 4.308 | 3.145 | 862 | 1258.000 |
|  | Lemon Heaven | CBD5-D | 8.86 | 2.817 | 3.145 | 563 | 1258.000 |
|  | Lemon Master | CBD6-D | 10.17 | 3.234 | 3.145 | 647 | 1258.000 |
|  | Fantasy Bud | CBD7-D | 8 | 2.544 | 3.145 | 509 | 1258.000 |
|  | Kosher Haze | THC1-D | 14 | 4.452 | 3.145 | 890 | 1258.000 |
|  | Prima Holandica | THC2-D | 17.3 | 5.501 | 3.145 | 1100 | 1258.000 |

Notes: *Working solution adjusted to have 10 mM concentration of the most abundant bioactive compound. CBD 1-7 and THC 1-2 plant extracts contained cannabidiolic and ∆9 -tetrahydrocannabinolic acids as the most abundant bioactive compound, respectively. while the extracts abbreviated with ‘D' represent decarboxylated samples and contained cannabidiol (CBD) or ∆9-tetrahydrocannabinol (∆9-THC) as the predominant cannabinoid Dilution factors of working and all final solutions refer to dilution of the original stock.

**Supplementary Table S2.** Dilutions of stock extract solutions for antiviral assays.

| **Extract** | **Genotype** | **Sample code** | **Stock solution**  **(mg/mL)** | **Dil. Factor** | ***Working solution (mg/mL)** | **Dil.**  **Factor** | **10.0 µM Final solution (µg/mL)** |
| --- | --- | --- | --- | --- | --- | --- | --- |
| Acid form | Elleta Campana | CBD1 | 14.4 | 4.017 | 3.585 | 4017 | 358.500 |
|  | Harlequin | CBD2 | 6.8 | 1.897 | 3.585 | 1897 | 358.500 |
|  | Strawberry | CBD3 | 9.75 | 2.72 | 3.585 | 2720 | 358.500 |
|  | Mandarin | CBD4 | 16.05 | 4.477 | 3.585 | 1605 | 358.500 |
|  | Lemon Heaven | CBD5 | 10.69 | 2.982 | 3.585 | 1069 | 358.500 |
|  | Lemon Master | CBD6 | 12.14 | 3.386 | 3.585 | 1214 | 358.500 |
|  | Fantasy Bud | CBD7 | 11.94 | 3.331 | 3.585 | 1194 | 358.500 |
|  | Kosher Haze | THC1 | 16.44 | 4.586 | 3.585 | 1644 | 358.500 |
|  | Prima Holandica | THC2 | 17.791 | 4.963 | 3.585 | 1779 | 358.500 |
| Neutral form | Elleta Campana | CBD1-D | 12.27 | 3.901 | 3.145 | 3901 | 314.500 |
|  | Harlequin | CBD2-D | 5.95 | 1.892 | 3.145 | 1892 | 314.500 |
|  | Strawberry | CBD3-D | 8.19 | 2.604 | 3.145 | 2604 | 314.500 |
|  | Mandarin | CBD4-D | 13.55 | 4.308 | 3.145 | 4308 | 314.500 |
|  | Lemon Heaven | CBD5-D | 8.86 | 2.817 | 3.145 | 2817 | 314.500 |
|  | Lemon Master | CBD6-D | 10.17 | 3.234 | 3.145 | 3234 | 314.500 |
|  | Fantasy Bud | CBD7-D | 8 | 2.544 | 3.145 | 2544 | 314.500 |
|  | Kosher Haze | THC1-D | 14 | 4.452 | 3.145 | 4452 | 314.500 |
|  | Prima Holandica | THC2-D | 17.3 | 5.501 | 3.145 | 5501 | 314.500 |

Notes: *Working solution adjusted to have 10 mM concentration of the most abundant bioactive compound. CBD 1-7 and THC 1-2 plant extracts contained cannabidiolic and ∆9- tetrahydrocannabinolic acids as the most abundant bioactive compound, respectively, while the extracts abbreviated with ‘D' represent decarboxylated samples and contained cannabidiol (CBD) or ∆9 -tetrahydrocannabinol (∆9-THC) as the predominant cannabinoid. Dilution factors of working and all final solutions refer to dilution of the original stock.

**Supplementary Table S3.** Phytocannabinoid levels (mg/mL) in the extracts of tested Cannabis genotypes.

| **Extract** | **Acidic form** | | | | | | | | | **Neutral form** | | | | | | | | |
| --- | --- | --- | --- | --- | --- | --- | --- | --- | --- | --- | --- | --- | --- | --- | --- | --- | --- | --- |
| **Genotype** | **Elleta Campana** | **Harlequin** | **Strawberry** | **Mandarin** | **Lemon Heaven** | **Lemon Master** | **Fantasy Bud** | **Kosher Haze** | **Prima Holandica** | **Elleta Campana** | **Harlequin** | **Strawberry** | **Mandarin** | **Lemon Heaven** | **Lemon Master** | **Fantasy Bud** | **Kosher Haze** | **Prima Holandica** |
| **Sample code** | **CBD1** | **CBD2** | **CBD3** | **CBD4** | **CBD5** | **CBD6** | **CBD7** | **THC1** | **THC2** | **CBD1-D** | **CBD2-D** | **CBD3-D** | **CBD4-D** | **CBD5-D** | **CBD6-D** | **CBD7-D** | **THC1-D** | **THC2-D** |
| CBDA | 14.40 | 6.80 | 9.75 | 16.05 | 10.69 | 12.14 | 10.75 | LOQ | LOQ | 2.17 | 0.98 | 1.40 | 2.59 | 1.81 | 1.57 | 3.10 | LLOQ | LLOQ |
|  | ±0.17 | ±0.14 | ±0.12 | ±0.38 | ±0.24 | ±0.35 | ±0.26 |  |  | ±0.02 | ±0.02 | ±0.02 | ±0.03 | ±0.01 | ±0.02 | ±0.03 |  |  |
| Δ9-THCA | 0.35 | 0.12 | 0.25 | 0.47 | 0.24 | 0.34 | 0.30 | 15.19 | 16.82 | LLOQ | LLOQ | LLOQ | LLOQ | LLOQ | LLOQ | 0.01 | 1.17 | 0.55 |
|  | ±0.00 | ±0.00 | ±0.00 | ±0.02 | ±0.01 | ±0.03 | ±0.01 | ±0.04 | ±0.50 |  |  |  |  |  |  | ±0.00 | ±0.01 | ±0.00 |
| CBDVA | 0.03 | 0.01 | 0.01 | 0.07 | 0.01 | 0.02 | 0.24 | LLOQ | LLOQ | LLOQ | LLOQ | LLOQ | LLOQ | LLOQ | LLOQ | 0.08 | LLOQ | LLOQ |
|  | ±0.00 | ±0.00 | ±0.00 | ±0.00 | ±0.00 | ±0.00 | ±0.00 |  |  |  |  |  |  |  |  | ±0.00 |  |  |
| THCVA | LLOQ | LLOQ | LLOQ | LLOQ | LLOQ | LLOQ | LLOQ | 0.09 | 0.08 | LLOQ | LLOQ | LLOQ | LLOQ | LLOQ | LLOQ | LLOQ | LLOQ | LLOQ |
|  |  |  |  |  |  |  |  | ±0.00 | ±0.00 | LLOQ | LLOQ | LLOQ | LLOQ | LLOQ | LLOQ | LLOQ |  |  |
| CBGA | 0.01 | LLOQ | LLOQ | 0.01 | LOQ | 0.01 | LOQ | 0.22 | 0.23 | LLOQ | LLOQ | LLOQ | LLOQ | LLOQ | LLOQ | LLOQ | 0.06 | 0.08 |
|  | ±0.00 |  |  | ±0.00 |  | ±0.00 |  | ±0.00 | ±0.01 |  |  |  |  |  |  |  | ±0.00 | ±0.00 |
| CBCA | 0.54 | 0.31 | 0.51 | 0.75 | 0.42 | 0.56 | 0.46 | 0.12 | 0.35 | 0.07 | 0.05 | 0.06 | 0.10 | 0.08 | 0.06 | 0.12 | 0.09 | 0.16 |
|  | ±0.00 | ±0.00 | ±0.00 | ±0.03 | ±0.02 | ±0.01 | ±0.01 | ±0.00 | ±0.01 | ±0.00 | ±0.00 | ±0.00 | ±0.00 | ±0.00 | ±0.00 | ±0.01 | ±0.00 | ±0.02 |
| CBD | 0.98 | 0.52 | 0.47 | 0.61 | 0.64 | 0.71 | 1.00 | LLOQ | LLOQ | 12.27 | 5.95 | 8.19 | 13.55 | 8.86 | 10.17 | 7.55 | 0.01 | 0.02 |
|  | ±0.01 | ±0.01 | ±0.01 | ±0.02 | ±0.02 | ±0.01 | ±0.01 |  |  | ±0.39 | ±0.04 | ±0.09 | ±0.25 | ±0.11 | ±0.10 | ±0.01 | ±0.00 | ±0.00 |
| Δ9-THC | 0.15 | 0.07 | 0.06 | 0.09 | 0.09 | 0.09 | 0.11 | 1.30 | 2.32 | 0.56 | 0.24 | 0.35 | 0.63 | 0.38 | 0.45 | 0.39 | 14.04 | 17.71 |
|  | ±0.00 | ±0.00 | ±0.00 | ±0.00 | ±0.00 | ±0.01 | ±0.00 | ±0.06 | ±0.31 | ±0.01 | ±0.01 | ±0.00 | ±0.00 | ±0.00 | ±0.01 | ±0.00 | ±0.14 | ±0.33 |
| CBG | 0.01 | LLOQ | 0.02 | 0.15 | 0.04 | 0.03 | 0.04 | 0.06 | 0.11 | 0.15 | 0.01 | 0.09 | 0.27 | 0.09 | 0.13 | 0.10 | 0.20 | 0.26 |
|  | ±0.00 |  | ±0.00 | ±0.01 | ±0.00 | ±0.00 | ±0.02 | ±0.00 | ±0.00 | ±0.01 | ±0.00 | ±0.00 | ±0.01 | ±0.01 | ±0.00 | ±0.00 | ±0.01 | ±0.01 |
| CBC | 0.08 | 0.04 | 0.04 | 0.06 | 0.06 | 0.05 | 0.04 | LLOQ | LLOQ | 0.44 | 0.16 | 0.37 | 0.60 | 0.32 | 0.40 | 0.36 | 0.10 | 0.29 |
|  | ±0.00 | ±0.00 | ±0.00 | ±0.00 | ±0.00 | ±0.00 | ±0.00 |  |  | ±0.01 | ±0.00 | ±0.00 | ±0.01 | ±0.00 | ±0.01 | ±0.00 | ±0.00 | ±0.00 |
| CBDV | LLOQ | LLOQ | LLOQ | LLOQ | LLOQ | LLOQ | LLOQ | LLOQ | LLOQ | 0.06 | 0.03 | 0.02 | 0.10 | LLOQ | 0.06 | 0.19 | LLOQ | LLOQ |
|  |  |  |  |  |  |  |  |  |  | ±0.00 | ±0.00 | ±0.00 | ±0.00 |  | ±0.00 | ±0.00 |  |  |
| THCV | LLOQ | LLOQ | LLOQ | LLOQ | LLOQ | LLOQ | LLOQ | LLOQ | LLOQ | LLOQ | LLOQ | LLOQ | LLOQ | LLOQ | LLOQ | LLOQ | 0.14 | 0.12 |
|  |  |  |  |  |  |  |  |  |  |  |  |  |  |  |  |  | ±0.01 | ±0.00 |

Notes: Abbreviations of compounds: CBDA, cannabidiolic acid; Δ9-THCA, Δ9-tetrahydrocannabinolic acid; CBDVA, cannabidivarinic acid; THCVA, Tetrahydrocannabivarinic acid; CGBA, cannabigerolic acid; CBCA, cannabichromenic acid; CBD, cannabidiol; Δ9-THC, Δ9-tetrahydrocannabinol; CBG, cannabigerol; CBC, cannabichromene; CBDV, cannabidivarin; THCV, Tetrahydrocannabivarin; <LLOQ, lower limit of quantification (<0.01 mg/mL).

**Supplementary Table S4.** Phenolic acid levels (ng/mL) in the extracts of tested Cannabis genotypes.

| **Extract** | **Acidic form** | | | | | | | | | **Neutral form** | | | | | | | | |
| --- | --- | --- | --- | --- | --- | --- | --- | --- | --- | --- | --- | --- | --- | --- | --- | --- | --- | --- |
| **Genotype** | **Elleta Campana** | **Harlequin** | **Strawberry** | **Man.d.arin** | **Lemon Heaven** | **Lemon Master** | **Fantasy Bud** | **Kosher Haze** | **Prima Holandica** | **Elleta Campana** | **Harlequin** | **Strawberry** | **Man.d.arin** | **Lemon Heaven** | **Lemon Master** | **Fantasy Bud** | **Kosher Haze** | **Prima Holandica** |
| **Sample code** | **CBD1** | **CBD2** | **CBD3** | **CBD4** | **CBD5** | **CBD6** | **CBD7** | **THC1** | **THC2** | **CBD1-D** | **CBD2-D** | **CBD3-D** | **CBD4-D** | **CBD5-D** | **CBD6-D** | **CBD7-D** | **THC1-D** | **THC2-D** |
| 34HBA | 5.48 | 8.19 | 17.62 | 9.78 | 5.81 | 3.28 | 20.35 | 18.18 | 23.47 | 0.13 | 0.10 | 0.30 | LOQ | 0.06 | LLOQ | 29.26 | 17.43 | 25.68 |
|  | ±2.22 | ±0.53 | ±1.42 | ±0.48 | ±0.57 | ±0.33 | ±1.01 | ±2.41 | ±1.40 | ±0.00 | ±0.01 | ±0.00 |  | ±0.01 |  | ±2.54 | ±2.11 | ±1.42 |
| SaAG | 383.54 | 501.03 | 618.78 | 228.80 | 204.32 | 302.31 | 32.38 |  |  | 0.73 | 0.87 | 1.06 | 0.36 | 0.31 | 0.71 | 40.76 | LLOQ | LLOQ |
|  | ±64.30 | ±19.24 | ±18.62 | ±3.49 | ±12.83 | ±69.49 | ±2.08 |  |  | ±0.01 | ±0.03 | ±0.07 | ±0.02 | ±0.01 | ±0.04 | ±2.79 |  |  |
| CGA | 5.78 | 4.60 | 11.79 | 10.48 | 4.15 | LLOQ | 22.75 | LLOQ | LLOQ | 0.03 | 0.03 | 0.04 | 0.04 | 0.02 | 0.03 | 16.88 | LLOQ | LLOQ |
|  | ±0.96 | ±0.90 | ±2.02 | ±1.01 | ±0.17 |  | ±0.31 |  |  | ±0.00 | ±0.00 | ±0.01 | ±0.01 | ±0.00 | ±0.00 | ±0.39 |  |  |
| VA | 82.17 | 87.06 | 51.90 | 43.91 | 72.73 | 63.14 | 8.09 | 79.51 | 65.61 | 1.35 | 1.28 | 0.97 | 0.61 | 0.99 | 1.00 | 13.29 | 48.62 | 46.32 |
|  | ±18.05 | ±5.08 | ±5.46 | ±1.63 | ±4.59 | ±11.90 | ±0.68 | ±11.53 | ±4.49 | ±0.02 | ±0.03 | ±0.03 | ±0.02 | ±0.03 | ±0.01 | ±1.03 | ±1.80 | ±3.31 |
| CA | LLOQ | LLOQ | LLOQ | LLOQ | LLOQ | LLOQ | 3.35 | 0.27 | 0.62 | 0.16 | 0.08 | 0.08 | 0.05 | LLOQ | LLOQ | 8.76 | 0.43 | 1.04 |
|  |  |  |  |  |  |  | ±0.19 | ±0.11 | ±0.11 | ±0.01 | ±0.00 | ±0.01 | ±0.00 |  |  | ±0.82 | ±0.02 | ±0.09 |
| SyA | LLOQ | LLOQ | LLOQ | LLOQ | LLOQ | LLOQ | 1.73 | LLOQ | LLOQ | 0.07 | 0.10 | 0.06 | 0.05 | 0.09 | 0.09 | 2.70 | LLOQ | LLOQ |
|  |  |  |  |  |  |  | ±0.22 |  |  | ±0.00 | ±0.01 | ±0.00 | ±0.01 | ±0.01 | ±0.00 | ±0.31 |  |  |
| 4HBA | 74.59 | 73.51 | 33.12 | 65.02 | 47.96 | 57.29 | 16.25 | 34.46 | 23.95 | 0.76 | 0.82 | 0.41 | 0.56 | 0.60 | 0.80 | 21.15 | 21.59 | 18.96 |
|  | ±10.31 | ±1.68 | ±2.44 | ±3.02 | ±3.60 | ±13.00 | ±1.13 | ±3.88 | ±1.67 | ±0.02 | ±0.01 | ±0.02 | ±0.01 | ±0.02 | ±0.02 | ±1.95 | ±0.98 | ±1.02 |
| *p*CA | 29.22 | 72.22 | 32.95 | 72.73 | 41.18 | 30.96 | 31.13 | 16.26 | 24.24 | 2.85 | 2.76 | 2.04 | 1.51 | 1.52 | 1.42 | 79.07 | 17.00 | 29.44 |
|  | ±4.50 | ±1.02 | ±1.49 | ±0.06 | ±4.09 | ±5.13 | ±0.63 | ±0.63 | ±1.52 | ±0.10 | ±0.07 | ±0.04 | ±0.09 | ±0.02 | ±0.06 | ±4.15 | ±0.74 | ±0.33 |
| SaA | 275.36 | 367.59 | 269.51 | 289.27 | 150.85 | 315.88 | 61.22 | 8.27 | 12.06 | 3.08 | 5.25 | 4.13 | 1.89 | 2.05 | 4.35 | 75.86 | 10.71 | 8.56 |
|  | ±13.19 | ±3.52 | ±6.63 | ±3.94 | ±9.56 | ±50.69 | ±1.18 | ±1.36 | ±0.18 | ±0.02 | ±0.09 | ±0.06 | ±0.03 | ±0.03 | ±0.18 | ±2.77 | ±0.88 | ±0.97 |
| FA | LLOQ | 18.29 | LLOQ | LLOQ | LLOQ | LLOQ | 11.32 | 15.45 | 11.83 | 0.13 | 0.27 | 0.11 | 0.07 | 0.12 | 0.12 | 20.94 | 15.53 | 19.18 |
|  |  | ±0.32 |  |  |  |  | ±0.31 | ±0.51 | ±0.81 | ±0.01 | ±0.01 | ±0.01 | ±0.00 | ±0.00 | ±0.00 | ±1.75 | ±0.61 | ±0.72 |
| SiA | LLOQ | 31.69 | LLOQ | 13.33 | 19.20 | 22.18 | LLOQ | LLOQ | LLOQ | 0.09 | 0.24 | 0.09 | 0.06 | 0.11 | 0.12 | LLOQ | LLOQ | LLOQ |
|  |  | ±1.89 |  | ±0.38 | ±2.74 | ±3.00 |  |  |  | ±0.00 | ±0.01 | ±0.01 | ±0.01 | ±0.00 | ±0.00 |  |  |  |

Notes: Abbreviations of compounds: 34DHBA, 3,4-dihydroxybenzoic acid; SaAG, salicylic acid glucoside; CGA, chlorogenic acid; VA, vanillic acid; CA, caffeic acid; SyA, syringic acid; 4HBA, 4-hydroxybenzoic acid; pCA, *p*-coumaric acid; SaA, salicylic acid; FA, ferulic acid; SiA, sinapic acid; <LOQ, values below the limit of quantification; <LLOQ, lower limit of quantification (<0.01 ng/mL).

**Supplementary Table S5.** Flavonoid levels (ng/mL) in extracts of tested Cannabis genotypes.

| **Extract** | **Acidic form** | | | | | | | | | **Neutral form** | | | | | | | | |
| --- | --- | --- | --- | --- | --- | --- | --- | --- | --- | --- | --- | --- | --- | --- | --- | --- | --- | --- |
| **Genotype** | **Elleta Campana** | **Harlequin** | **Strawberry** | **Mandarin** | **Lemon Heaven** | **Lemon Master** | **Fantasy Bud** | **Kosher Haze** | **Prima Holandica** | **Elleta Campana** | **Harlequin** | **Strawberry** | **Mandarin** | **Lemon Heaven** | **Lemon Master** | **Fantasy Bud** | **Kosher Haze** | **Prima Holandica** |
| **Sample code** | **CBD1** | **CBD2** | **CBD3** | **CBD4** | **CBD5** | **CBD6** | **CBD7** | **THC1** | **THC2** | **CBD1-D** | **CBD2-D** | **CBD3-D** | **CBD4-D** | **CBD5-D** | **CBD6-D** | **CBD7-D** | **THC1-D** | **THC2-D** |
| ORI | 223.42 | 801.98 | 8.10 | 223.56 | 626.26 | 492.13 | 7.31 | 256.49 | 357.89 | 830.11 | 2962.24 | 5.91 | 491.34 | 1781.38 | 1629.81 | 12.75 | 133.45 | 264.28 |
|  | ±12.32 | ±50.41 | ±1.09 | ±16.35 | ±49.69 | ±74.93 | ±0.46 | ±27.13 | ±3.47 | ±76.52 | ±34.38 | ±0.36 | ±18.21 | ±67.28 | ±76.05 | ±0.67 | ±12.75 | ±37.85 |
| VIT | 6.90 | 235.26 | LLOQ | 4.38 | 105.65 | 94.55 | 327.33 | 11.68 | 29.41 | 0.16 | 3.91 | LLOQ | LLOQ | 1.37 | 2.77 | 509.13 | 6.64 | 16.21 |
|  | ±1.13 | ±15.63 |  | ±0.38 | ±14.03 | ±22.66 | ±18.01 | ±0.94 | ±4.71 | ±0.02 | ±0.09 |  |  | ±0.05 | ±0.08 | ±9.41 | ±0.23 | ±1.15 |
| ABI | 3.28 | 4.93 | 3.68 | 4.00 | 5.07 | 3.90 | 4.98 | LLOQ | LLOQ | 0.06 | 0.08 | 0.08 | 0.03 | 0.06 | 0.06 | 7.53 | LLOQ | LLOQ |
|  | ±0.49 | ±0.59 | ±0.50 | ±0.10 | ±0.41 | ±0.90 | ±0.19 |  |  | ±0.00 | ±0.00 | ±0.00 | ±0.00 | ±0.00 | ±0.00 | ±0.40 |  |  |
| RUT | 173.37 | 230.13 | 508.38 | LLOQ | 387.88 | 139.60 | 31.74 | 81.69 | 36.07 | 1.35 | 1.75 | 3.45 | LLOQ | 2.70 | 1.16 | 45.44 | 35.24 | 27.76 |
|  | ±6.45 | ±2.98 | ±23.15 |  | ±17.11 | ±15.31 | ±1.34 | ±6.01 | ±1.80 | ±0.05 | ±0.05 | ±0.13 |  | ±0.01 | ±0.07 | ±2.69 | ±5.84 | ±2.09 |
| ERI | LLOQ | 5.41 | LLOQ | LLOQ | LLOQ | 2.30 | 0.24 | LLOQ | LLOQ | LLOQ | 0.04 | LLOQ | LLOQ | LLOQ | LLOQ | 0.24 | LLOQ | LLOQ |
|  |  | ±0.13 |  |  |  | ±0.11 | ±0.00 |  |  |  | ±0.00 |  |  |  |  | ±0.02 |  |  |
| LUT | 180.67 | 1244.32 | 958.01 | 236.31 | 864.20 | 697.74 | 191.47 | 49.88 | 67.73 | 1.31 | 7.44 | 3.32 | 0.28 | 1.39 | 1.05 | 228.68 | 124.93 | 158.85 |
|  | ±29.49 | ±86.87 | ±89.89 | ±16.85 | ±37.93 | ±73.53 | ±21.36 | ±3.48 | ±9.45 | ±0.07 | ±0.57 | ±0.58 | ±0.02 | ±0.18 | ±0.03 | ±27.29 | ±19.55 | ±10.15 |
| API | LLOQ | LLOQ | LLOQ | LLOQ | LLOQ | LLOQ | LLOQ | LLOQ | LLOQ | LLOQ | 0.09 | LLOQ | LLOQ | LLOQ | LLOQ | LLOQ | LLOQ | LLOQ |
|  |  |  |  |  |  |  |  |  |  |  | ±0.01 |  |  |  |  |  |  |  |
| CANN B | LLOQ | 98.79 | 44.01 | 37.63 | 65.47 | 86.80 | 37.34 | 14.85 | 62.69 | 2.07 | 2.98 | 1.07 | 0.85 | 2.66 | 1.75 | 89.30 | 35.12 | 106.75 |
|  |  | ±6.83 | ±5.87 | ±6.87 | ±2.87 | ±5.82 | ±6.42 | ±1.43 | ±1.87 | ±0.29 | ±0.24 | ±0.08 | ±0.08 | ±0.32 | ±0.08 | ±9.84 | ±3.99 | ±7.68 |
| CANN A | 212.30 | 448.82 | 901.15 | 659.14 | 818.04 | 223.52 | 419.43 | 29.43 | 128.63 | 7.78 | 8.39 | 7.64 | 6.41 | 9.87 | 7.68 | 624.53 | 92.35 | 164.81 |
|  | ±37.83 | ±84.19 | ±43.14 | ±11.23 | ±14.44 | ±11.71 | ±43.56 | ±0.24 | ±5.80 | ±1.07 | ±0.37 | ±0.28 | ±0.37 | ±0.61 | ±1.27 | ±43.00 | ±8.15 | ±3.39 |
| QUE | LLOQ | 38.70 | LLOQ | LLOQ | LLOQ | LLOQ | 5.65 | 0.93 | 2.22 | LLOQ | LLOQ | LLOQ | LLOQ | LLOQ | LLOQ | 6.53 | 1.15 | 1.06 |
|  |  | ±2.12 |  |  |  |  | ±0.84 | ±0.15 | ±0.22 |  |  |  |  |  |  | ±0.68 | ±0.06 | ±0.22 |
| CHR | LLOQ | LLOQ | LLOQ | LLOQ | LLOQ | LLOQ | 0.05 | 0.80 | 1.01 | LLOQ | LLOQ | LLOQ | LLOQ | LLOQ | LLOQ | 0.03 | 0.46 | 0.83 |
|  |  |  |  |  |  |  | ±0.01 | ±0.09 | ±0.14 |  |  |  |  |  |  | ±0.00 | ±0.09 | ±0.09 |

Notes: Abbreviations of compounds: ORI, orientin; VIT, vitexin; ABI, abietin; RUT, rutin; ERI, eriodyctiol; LUT, luteolin; API, apigenin; CANN B, cannflavin B; CANN A, cannflavin A; QUE, quercetin; CHR, chrysoeriol; <LLOQ, values below the limit of detection (<0.01 ng/mL).

**Supplementary Table S6.** Terpenoid profiles (%) in extracts of tested Cannabis genotypes.

| **Extract** | **Acidic form** | | | | | | | | | **Neutral form** | | | | | | | | |
| --- | --- | --- | --- | --- | --- | --- | --- | --- | --- | --- | --- | --- | --- | --- | --- | --- | --- | --- |
| **Genotype** | **Elleta Campana** | **Harlequin** | **Strawberry** | **Mandarin** | **Lemon Heaven** | **Lemon Master** | **Fantasy Bud** | **Kosher Haze** | **Prima Holandica** | **Elleta Campana** | **Harlequin** | **Strawberry** | **Mandarin** | **Lemon Heaven** | **Lemon Master** | **Fantasy Bud** | **Kosher Haze** | **Prima Holandica** |
| **Sample** | **CBD1** | **CBD2** | **CBD3** | **CBD4** | **CBD5** | **CBD6** | **CBD7** | **THC1** | **THC2** | **CBD1-D** | **CBD2-D** | **CBD3-D** | **CBD4-D** | **CBD5-D** | **CBD6-D** | **CBD7-D** | **THC1-D** | **THC2-D** |
| Myr | 5.8 | 1.2 | 0.7 | 0.5 | 0.4 | 0.9 | 0.6 | 1.6 | 2.1 | 5.5 | 1.7 | 0.9 | 0.6 | 0.8 | 0.7 | n.d. | 1.2 | 2.0 |
|  | ±0.1 | ±0.0 | ±0.0 | ±0.0 | ±0.0 | ±0.0 | ±0.0 | ±0.0 | ±0.1 | ±0.1 | ±0.0 | ±0.0 | ±0.0 | ±0.0 | ±0.0 |  | ±0.1 | ±0.0 |
| Lin | 1.4 | 0.4 | 0.5 | 0.7 | 0.2 | 0.3 | n.d. | 1.1 | 1.0 | 1.2 | 0.5 | 0.6 | 0.9 | 0.2 | 0.2 | n.d. | 0.9 | 1.0 |
|  | ±0.0 | ±0.0 | ±0.0 | ±0.0 | ±0.0 | ±0.0 |  | ±0.0 | ±0.1 | ±0.0 | ±0.0 | ±0.0 | ±0.0 | ±0.0 | ±0.0 |  | ±0.1 | ±0.0 |
| Fen | 0.6 | 0.3 | 0.2 | 0.2 | 0.3 | 0.3 | n.d. | n.d. | n.d. | 0.6 | 0.3 | 0.2 | 0.2 | 0.2 | 0.3 | n.d. | n.d. | n.d. |
|  | ±0.0 | ±0.0 | ±0.0 | ±0.0 | ±0.0 | ±0.0 |  |  |  | ±0.0 | ±0.0 | ±0.0 | ±0.0 | ±0.1 | ±0.0 |  |  |  |
| Lin | 5.8 | 0.6 | 5.5 | 6.8 | 4.1 | 3.2 | 0.6 | n.d. | 4.2 | 6.7 | 0.8 | 5.5 | 6.9 | 3.2 | 2.9 | 0.6 | n.d. | n.d. |
|  | ±0.1 | ±0.0 | ±0.1 | ±0.0 | ±0.0 | ±0.0 | ±0.0 |  | ±0.1 | ±0.0 | ±0.0 | ±0.3 | ±0.1 | ±0.0 | ±0.1 | ±0.0 |  |  |
| aFen | 3.4 | 2.0 | 1.5 | 2.3 | 1.4 | 1.6 | 0.7 | 1.4 | 2.3 | 3.2 | 2.0 | 1.6 | 2.4 | 1.3 | 1.5 | 0.6 | 1.2 | 2.1 |
|  | ±0.0 | ±0.0 | ±0.0 | ±0.0 | ±0.0 | ±0.0 | ±0.0 | ±0.0 | ±0.1 | ±0.0 | ±0.0 | ±0.0 | ±0.0 | ±0.0 | ±0.0 | ±0.0 | ±0.0 | ±0.0 |
| tPiH | 2.6 | 1.6 | 1.2 | 1.6 | 1.2 | 1.3 | n.d. | 1.1 | 1.8 | n.d. | 1.2 | 0.9 | 1.3 | 0.8 | 0.9 | n.d. | 0.8 | 1.4 |
|  | ±0.0 | ±0.0 | ±0.0 | ±0.0 | ±0.0 | ±0.0 |  | ±0.0 | ±0.0 |  | ±0.0 | ±0.0 | ±0.0 | ±0.0 | ±0.0 |  | ±0.0 | ±0.0 |
| Ips | 1.8 | 0.4 | n.d. | 0.1 | 0.3 | 0.3 | 0.4 | n.d. | n.d. | 1.3 | 0.4 | n.d. | 0.1 | 0.2 | 0.3 | 0.3 | n.d. | n.d. |
|  | ±0.1 | ±0.0 |  | ±0.0 | ±0.0 | ±0.0 | ±0.0 |  |  | ±0.0 | ±0.0 |  | ±0.0 | ±0.0 | ±0.0 | ±0.0 |  |  |
| Bor | 1.3 | 0.8 | 0.6 | 0.8 | 0.7 | 0.8 | 0.3 | 0.4 | 0.8 | 1.4 | 0.8 | 0.6 | 0.8 | 0.7 | 0.8 | 0.4 | 0.4 | 0.8 |
|  | ±0.0 | ±0.0 | ±0.0 | ±0.0 | ±0.0 | ±0.0 | ±0.0 | ±0.1 | ±0.0 | ±0.0 | ±0.0 | ±0.0 | ±0.0 | ±0.0 | ±0.0 | ±0.0 | ±0.0 | ±0.0 |
| aTer | 4.2 | 2.4 | 1.8 | 2.1 | 2.0 | 2.0 | 0.8 | 1.3 | 2.3 | 4.0 | 2.3 | 1.8 | 2.3 | 1.9 | 2.1 | 0.8 | 1.2 | 2.2 |
|  | ±0.0 | ±0.0 | ±0.0 | ±0.0 | ±0.1 | ±0.0 | ±0.0 | ±0.0 | ±0.0 | ±0.0 | ±0.0 | ±0.0 | ±0.0 | ±0.0 | ±0.0 | ±0.0 | ±0.0 | ±0.0 |
| HBut | n.d. | n.d. | n.d. | 1.0 | n.d. | n.d. | n.d. | n.d. | 0.6 | n.d. | n.d. | n.d. | 1.1 | n.d. | n.d. | n.d. | n.d. | 0.6 |
|  |  |  |  | ±0.0 |  |  |  |  | ±0.0 |  |  |  | ±0.0 |  |  |  |  | ±0.0 |
| Cit | 3.2 | 0.3 | n.d. | n.d. | n.d. | n.d. | 0.4 | n.d. | 0.9 | 3.1 | 0.3 | n.d. | n.d. | 0.3 | 0.4 | 0.4 | n.d. | 1.0 |
|  | ±0.1 | ±0.0 |  |  |  |  | ±0.0 |  | ±0.0 | ±0.0 | ±0.0 |  |  | ±0.0 | ±0.0 | ±0.0 |  | ±0.0 |
| HHex | n.d. | n.d. | n.d. | n.d. | n.d. | n.d. | n.d. | 0.4 | 1.7 | n.d. | n.d. | n.d. | 0.3 | n.d. | n.d. | n.d. | 0.4 | 1.7 |
|  |  |  |  |  |  |  |  | ±0.0 | ±0.0 |  |  |  | ±0.0 |  |  |  | ±0.0 | ±0.0 |
| bCar | 8.5 | 18.0 | 13.1 | 10.5 | 7.6 | 4.8 | 10.0 | 10.5 | 10.8 | 8.5 | 18.0 | 13.1 | 10.6 | 7.0 | 4.8 | 10.2 | 11.0 | 12.1 |
|  | ±0.1 | ±0.1 | ±0.1 | ±0.0 | ±0.2 | ±0.0 | ±0.0 | ±0.0 | ±0.1 | ±0.1 | ±0.2 | ±0.1 | ±0.0 | ±0.1 | ±0.0 | 0.0 | ±0.1 | ±0.1 |
| gEle | n.d. | n.d. | n.d. | n.d. | n.d. | n.d. | n.d. | 3.1 | 1.4 | n.d. | n.d. | n.d. | n.d. | n.d. | n.d. | n.d. | 2.6 | 1.3 |
|  |  |  |  |  |  |  |  | ±0.1 | ±0.1 |  |  |  |  |  |  |  | ±0.0 | ±0.0 |
| tBerg | 0.5 | 0.6 | 1.3 | 3.2 | 0.5 | 0.3 | 0.8 | 3.0 | 2.7 | 0.5 | 0.6 | 1.4 | 3.4 | 0.5 | 0.4 | 0.7 | 2.8 | 2.6 |
|  | ±0.0 | ±0.0 | ±0.0 | ±0.0 | ±0.0 | ±0.0 | ±0.0 | ±0.0 | ±0.1 | ±0.0 | ±0.1 | ±0.0 | ±0.0 | ±0.0 | ±0.0 | 0.1 | ±0.0 | ±0.0 |
| aHum | 2.6 | 5.4 | 4.1 | 5.3 | 2.4 | 1.5 | 5.2 | 4.4 | 4.7 | 2.7 | 5.4 | 4.2 | 5.4 | 2.2 | 1.6 | 5.5 | 4.6 | 5.2 |
|  | ±0.0 | ±0.0 | ±0.0 | ±0.0 | ±0.0 | ±0.0 | ±0.0 | ±0.0 | ±0.0 | ±0.0 | ±0.1 | ±0.0 | ±0.0 | ±0.0 | ±0.1 | 0.0 | ±0.0 | ±0.1 |
| bFar | 1.0 | 1.0 | 2.0 | 4.5 | 1.1 | 0.7 | 0.2 | 3.8 | 3.8 | 1.0 | 1.0 | 1.9 | 4.3 | 1.0 | 0.8 | n.d. | 3.1 | 3.5 |
|  | ±0.0 | ±0.0 | ±0.0 | ±0.0 | ±0.0 | ±0.0 | ±0.0 | ±0.0 | ±0.0 | ±0.0 | ±0.0 | ±0.1 | ±0.0 | ±0.0 | ±0.0 |  | ±0.0 | ±0.0 |
| gMur | n.d. | n.d. | n.d. | n.d. | n.d. | n.d. | n.d. | 1.2 | 0.9 | n.d. | n.d. | n.d. | n.d. | n.d. | n.d. | n.d. | 1.1 | 0.8 |
|  |  |  |  |  |  |  |  | ±0.0 | ±0.0 |  |  |  |  |  |  |  | ±0.0 | ±0.0 |
| bSel | 0.5 | n.d. | n.d. | 0.3 | n.d. | 0.8 | 3.0 | 3.9 | 3.2 | 0.6 | n.d. | n.d. | n.d. | n.d. | n.d. | 2.8 | 3.4 | 2.9 |
|  | ±0.0 |  |  | ±0.0 |  | ±0.0 | ±0.0 | ±0.1 | ±0.0 | ±0.0 |  |  |  |  |  | 0.0 | ±0.0 | ±0.0 |
| aSel | 0.3 | n.d. | n.d. | n.d. | n.d. | 0.4 | 2.5 | 3.7 | 3.0 | 0.3 | n.d. | n.d. | n.d. | n.d. | 0.4 | 2.5 | 3.5 | 2.9 |
|  | ±0.0 |  |  |  |  | ±0.0 | ±0.0 | ±0.0 | ±0.0 | ±0.0 |  |  |  |  | ±0.0 | 0.0 | ±0.1 | ±0.0 |
| bBis | 4.6 | 2.9 | 2.8 | 1.5 | 2.2 | 1.5 | n.d. | 0.6 | 0.5 | 4.0 | 2.8 | 2.4 | 1.2 | 2.0 | 1.5 | n.d. | 0.4 | n.d. |
|  | ±0.0 | ±0.0 | ±0.1 | ±0.0 | ±0.0 | ±0.0 |  | ±0.0 | ±0.0 | ±0.0 | ±0.0 | ±0.0 | ±0.0 | ±0.0 | ±0.0 |  | ±0.0 |  |
| bCur | n.d. | 0.4 | 0.7 | 0.8 | 0.4 | 0.3 | n.d. | 0.5 | 0.6 | n.d. | 0.4 | 0.8 | 1.1 | 0.4 | 0.3 | n.d. | 0.6 | 0.8 |
|  |  | ±0.1 | ±0.0 | ±0.1 | ±0.0 | ±0.0 |  | ±0.0 | ±0.0 |  | ±0.1 | ±0.0 | ±0.0 | ±0.1 | ±0.1 |  | ±0.0 | ±0.0 |
| dCad | n.d. | n.d. | n.d. | n.d. | n.d. | n.d. | n.d. | 2.1 | 1.5 | n.d. | n.d. | n.d. | n.d. | n.d. | n.d. | n.d. | 2.6 | 2.0 |
|  |  |  |  |  |  |  |  | ±0.1 | ±0.1 |  |  |  |  |  |  |  | ±0.1 | ±0.4 |
| bSes | 0.3 | 0.4 | 0.3 | 0.9 | 0.2 | 0.6 | n.d. | 1.7 | 1.4 | 0.3 | 0.4 | 0.4 | 0.9 | 0.3 | 0.6 | n.d. | 1.8 | 1.5 |
|  | ±0.0 | ±0.0 | ±0.0 | ±0.0 | ±0.2 | ±0.0 |  | ±0.0 | ±0.0 | ±0.0 | ±0.0 | ±0.0 | ±0.0 | ±0.0 | ±0.0 |  | ±0.0 | ±0.0 |
| Sel47D | n.d. | n.d. | n.d. | n.d. | n.d. | 4.3 | n.d. | 8.0 | 5.1 | n.d. | n.d. | n.d. | n.d. | n.d. | 3.2 | n.d. | 11.1 | 7.1 |
|  |  |  |  |  |  | ±0.1 |  | ±0.2 | ±0.1 |  |  |  |  |  | ±0.1 |  | ±0.3 | ±0.2 |
| Eud57D | 0.3 | n.d. | n.d. | n.d. | n.d. | 0.4 | n.d. | 2.3 | 2.2 | 0.4 | n.d. | n.d. | n.d. | n.d. | 0.2 | n.d. | n.d. | 2.9 |
|  | ±0.0 |  |  |  |  | ±0.0 |  | ±0.2 | ±0.1 | ±0.0 |  |  |  |  | ±0.0 |  |  | ±0.2 |
| Sel37D | n.d. | n.d. | n.d. | n.d. | n.d. | 3.6 | n.d. | 6.4 | 4.4 | n.d. | n.d. | n.d. | n.d. | n.d. | 1.9 | n.d. | 10.9 | 6.5 |
|  |  |  |  |  |  | ±0.1 |  | ±0.4 | ±0.1 |  |  |  |  |  | ±0.2 |  | ±1.5 | ±0.3 |
| aBis | 2.8 | 4.3 | 2.1 | 1.4 | 3.1 | 2.6 | 1.9 | 1.7 | 1.4 | 2.7 | 4.1 | 2.1 | 1.5 | 3.0 | 2.7 | 2.1 | 1.5 | 1.2 |
|  | ±0.0 | ±0.1 | ±0.1 | ±0.0 | ±0.1 | ±0.2 | ±0.0 | ±0.2 | ±0.0 | ±0.0 | ±0.0 | ±0.0 | ±0.0 | ±0.0 | ±0.2 | ±0.0 | ±0.0 | ±0.0 |
| GerB | n.d. | n.d. | n.d. | n.d. | n.d. | n.d. | n.d. | 1.8 | 0.9 | n.d. | n.d. | n.d. | n.d. | n.d. | n.d. | n.d. | 1.7 | 1.1 |
|  |  |  |  |  |  |  |  | ±0.1 | ±0.1 |  |  |  |  |  |  |  | ±0.1 | ±0.0 |
| Ner | 0.9 | n.d. | 1.4 | 0.6 | 0.7 | 0.5 | 1.0 | n.d. | 3.2 | 1.0 | 0.2 | 1.4 | 0.7 | 0.8 | 0.8 | 0.9 | 2.1 | 3.1 |
|  | ±0.0 | n.d. | ±0.0 | ±0.0 | ±0.0 | ±0.2 | ±0.0 |  | ±0.1 | ±0.0 | ±0.0 | ±0.0 | ±0.0 | ±0.0 | ±0.0 | ±0.0 | ±0.0 | ±0.0 |
| CarO | 1.7 | 3.1 | 1.2 | 1.4 | 1.7 | 1.1 | 7.3 | 4.0 | 4.9 | 1.4 | 2.5 | 1.2 | 1.4 | 1.3 | 0.9 | 5.7 | 2.3 | 2.6 |
|  | ±0.0 | ±0.0 | ±0.0 | ±0.0 | ±0.0 | ±0.0 | ±0.0 | ±0.0 | ±0.1 | ±0.0 | ±0.0 | ±0.0 | ±0.0 | ±0.0 | ±0.0 | ±0.0 | ±0.0 | ±0.0 |
| Gui | 7.3 | 7.7 | 10.9 | 11.3 | 11.6 | 10.9 | 13.5 | n.d. | n.d. | 7.4 | 7.5 | 10.9 | 10.8 | 11.7 | 10.6 | 15.0 | n.d. | n.d. |
|  | ±0.0 | ±0.1 | ±0.1 | ±0.1 | ±0.2 | ±0.1 | ±0.1 |  |  | ±0.0 | ±0.0 | ±0.1 | ±0.1 | ±0.0 | ±0.2 | ±0.0 |  |  |
| 57Eud | 0.5 | 0.4 | 1.2 | 0.9 | 1.2 | 1.0 | n.d. | n.d. | n.d. | 0.3 | 0.4 | 1.0 | 1.0 | 1.6 | 1.4 | n.d. | n.d. | n.d. |
|  | ±0.0 | ±0.0 | ±0.5 | ±0.0 | ±0.6 | ±0.2 |  |  |  | ±0.0 | ±0.0 | ±0.5 | ±0.1 | ±0.6 | ±0.0 |  |  |  |
| 10 Eud | 7.9 | 8.8 | 10.5 | 11.3 | 12.2 | 11.1 | 13.5 | 2.8 | 2.8 | 8.0 | 8.7 | 10.5 | 10.2 | 12.1 | 10.9 | 14.9 | 2.3 | 2.6 |
|  | ±0.1 | ±0.1 | ±0.1 | ±0.1 | ±0.1 | ±0.1 | ±0.1 | ±0.1 | ±0.1 | ±0.1 | ±0.0 | ±0.1 | ±0.0 | ±0.0 | ±0.1 | ±0.0 | ±0.0 | ±0.0 |
| Ere | 0.4 | n.d. | 1.0 | 1.3 | 0.6 | n.d. | n.d. | n.d. | n.d. | 0.5 | 0.4 | 1.0 | 1.4 | 0.7 | 2.5 | n.d. | n.d. | n.d. |
|  | ±0.0 | n.d. | ±0.0 | ±0.0 | ±0.0 |  |  |  |  | ±0.0 | ±0.0 | ±0.0 | ±0.1 | ±0.0 | ±0.0 |  |  |  |
| gEud | 1.4 | 1.3 | 2.1 | 1.9 | 2.0 | 2.3 | 3.0 | 1.3 | 1.4 | 1.5 | 1.4 | 2.1 | 1.9 | 2.0 | 2.4 | 3.2 | 1.0 | 1.1 |
|  | ±0.0 | ±0.0 | ±0.1 | ±0.0 | ±0.0 | ±0.0 | ±0.0 | ±0.0 | ±0.0 | ±0.0 | ±0.0 | ±0.0 | ±0.0 | ±0.0 | ±0.1 | ±0.0 | ±0.0 | ±0.1 |
| Amo | 0.4 | 0.6 | 0.6 | 0.6 | 0.8 | 0.6 | 1.2 | n.d. | n.d. | 0.5 | 0.8 | 0.7 | 1.0 | 1.0 | 0.8 | 1.1 | n.d. | n.d. |
|  | ±0.0 | ±0.0 | ±0.0 | ±0.0 | ±0.0 | ±0.0 | ±0.0 |  |  | ±0.0 | ±0.0 | ±0.0 | ±0.0 | ±0.1 | ±0.0 | ±0.0 |  |  |
| bEud | 4.2 | 4.0 | 6.1 | 5.2 | 6.4 | 6.1 | 8.2 | n.d. | n.d. | 4.3 | 4.0 | 6.2 | 5.8 | 6.8 | 6.6 | 8.7 | n.d. | 2.7 |
|  | ±0.1 | ±0.1 | ±0.0 | ±0.1 | ±0.1 | ±0.0 | ±0.1 |  |  | ±0.0 | ±0.1 | ±0.0 | ±0.1 | ±0.0 | ±0.1 | ±0.0 |  | ±0.1 |
| aEud | 4.5 | 4.0 | 6.3 | 5.7 | 6.4 | 6.8 | 9.0 | 3.6 | 2.7 | 4.6 | 4.2 | 6.5 | 6.2 | 6.7 | 7.5 | 9.7 | 3.4 | n.d. |
|  | ±0.0 | ±0.4 | ±0.1 | ±0.1 | ±0.1 | ±0.1 | ±0.1 | ±0.1 | ±0.4 | ±0.0 | ±0.1 | ±0.1 | ±0.0 | ±0.1 | ±0.1 | ±0.0 | ±0.1 |  |
| 7aEuD | 0.6 | 0.7 | 0.8 | 1.0 | 1.0 | 0.9 | 1.4 | 1.8 | 1.4 | 0.6 | 0.8 | 0.7 | 1.0 | 1.0 | 0.8 | 1.1 | 1.6 | 1.3 |
|  | ±0.0 | ±0.1 | ±0.0 | ±0.0 | ±0.0 | ±0.0 | ±0.0 | ±0.0 | ±0.0 | ±0.0 | ±0.0 | ±0.1 | ±0.0 | ±0.0 | ±0.2 | ±0.0 | ±0.1 | ±0.0 |
| Bul | 1.1 | 1.4 | 2.1 | 2.2 | 1.3 | 1.7 | 1.2 | n.d. | n.d. | 1.6 | 1.1 | 1.7 | 0.2 | 0.5 | 0.4 | 1.1 | n.d. | n.d. |
|  | ±0.1 | ±0.1 | ±0.3 | ±0.3 | ±0.3 | ±0.1 | ±0.0 |  |  | ±0.1 | ±0.1 | ±0.3 | ±0.0 | ±0.0 | ±0.1 | ±0.0 |  |  |
| aBis | 8.6 | 18.0 | 9.8 | 1.1 | 13.7 | 11.6 | 1.3 | 3.0 | 3.3 | 8.7 | 18.1 | 7.1 | 1.0 | 14.6 | 12.2 | 1.5 | 3.4 | 3.9 |
|  | ±0.0 | ±0.2 | ±0.1 | ±0.3 | ±0.1 | ±0.1 | ±0.0 | ±0.3 | ±0.0 | ±0.1 | ±0.1 | ±1.3 | ±0.0 | ±0.0 | ±0.1 | ±0.0 | ±0.0 | ±0.0 |
| nCeo | n.d. | n.d. | n.d. | n.d. | n.d. | n.d. | n.d. | 1.0 | 1.1 | n.d. | n.d. | n.d. | n.d. | n.d. | n.d. | n.d. | 1.7 | 1.7 |
|  |  |  |  |  |  |  |  | ±0.1 | ±0.0 |  |  |  |  |  |  |  | ±0.1 | ±0.1 |
| Farn | n.d. | n.d. | n.d. | n.d. | n.d. | n.d. | 0.9 | 0.4 | 0.8 | n.d. | n.d. | n.d. | n.d. | n.d. | n.d. | 1.0 | 0.4 | 0.9 |
|  |  |  |  |  |  |  | ±0.0 | ±0.0 | ±0.0 |  |  |  |  |  |  | ±0.0 | ±0.0 | ±0.0 |
| *Total identified* | *91.0* | *93.1* | *92.4* | *88.9* | *87.7* | *87.1* | *89.0* | *83.8* | *87.5* | *88.8* | *92.9* | *89.3* | *88.1* | *86.8* | *86.2* | *91.0* | *87.0* | *86.9* |
|  | *±0.1* | *±0.7* | *±0.8* | *±0.3* | *±0.4* | *±0.6* | *±0.5* | *±0.3* | *0.2* | *±0.1* | *±0.3* | *±5.2* | *±0.1* | *±0.6* | *±1.2* | *±0.2* | *±2.1* | *±0.4* |

Notes: Abbreviations of compounds: Myr, myrcene; Lim, limonene; Fen, fenchone; Lin, linalool; eFen, *endo*-fenchol; tPiH, *trans*-pinene hydrate; Ips, ipsdienol; Bor, borneol; aTer, α-terpineol; HBut, hexyl butanoate; Cit, citronellol; HHex, Hexyl hexanoate; bCar, β-caryophyllene; gEle, γ-elemene; tBerg, α-*trans*-bergamotene; aHum, α-humulene; bFar, (*E*)-β-farnesene; gMur, γ-muurolene; bSel, β-selinene; aSel, α-selinene; ZaBis, (*Z*)-α-bisabolene; bCur, β-curcumene; dCad, δ-cadinene; bSes, β-sesquiphellandrene; Sel47D, selina-4(15),7(11)-diene; EudD, eudesma-5,7(11)-diene; Sel37D, selina-3,7(11)-diene; EaBis, (*E*)-α-bisabolene; Ger, germacrene B; Ner, (*E*)-nerolidol; CarO, caryophyllene oxide; Gua, guaiol; 57Eud, 5-*epi*-7-*epi*-α-eudesmol; 10Eud, 10-*epi*-γ-eudesmol; Ere, eremoligenol; gEud, γ-eudesmol; Amo, amorph-4-en-10α-ol; bEud, β-eudesmol; aEud, α-eudesmol; 7aEud, 7-epi-α-eudesmol; Bul, bulnesol; aBis, α-bisabolol; nCed, 5-*neo*-cedranol, Farn, (2*E*, 6*Z*)-farnesol; n.d., not detected.

**Supplementary Table S7.** The IC_50_ values of plant extracts and single compounds against Vero 6 cells.

| **Extract** | **Genotype** | **Sample code** | **IC_50_ (µM**) |
| --- | --- | --- | --- |
| Acid form | Elleta Campana | CBD1 | 126.23 ± 12.19 |
|  | Harlequin | CBD2 | 151.50 ± 27.06 |
|  | Strawberry | CBD3 | 157.47 ± 23.04 |
|  | Mandarin | CBD4 | 166.03 ± 36.74 |
|  | Lemon Heaven | CBD5 | 162.33 ± 36.85 |
|  | Lemon Master | CBD6 | 161.43 ± 27.63 |
|  | Fantasy Bud | CBD7 | 164.80 ± 18.41 |
|  | Kosher Haze | THC1 | 88.54 ± 17.45 |
|  | Prima Holandica | THC2 | 87.96 ± 20.64 |
| Neutral form | Elleta Campana | CBD1-D | 40.33 ± 9.06 |
|  | Harlequin | CBD2-D | 38.34 ± 2.10 |
|  | Strawberry | CBD3-D | 37.60 ± 2.41 |
|  | Mandarin | CBD4-D | 41.94 ± 1.25 |
|  | Lemon Heaven | CBD5-D | 40.15 ± 0.90 |
|  | Lemon Master | CBD6-D | 40.19 ± 1.96 |
|  | Fantasy Bud | CBD7-D | 40.37 ± 3.32 |
|  | Kosher Haze | THC1-D | 54.60 ± 9.43 |
|  | Prima Holandica | THC2-D | 56.40 ± 7.00 |
| Standard |  | CBD | > 25.00 |
|  |  | CBDA | > 25.00 |
|  |  | Δ9-THC | > 25.00 |
|  |  | Δ9-THCA | > 25.00 |


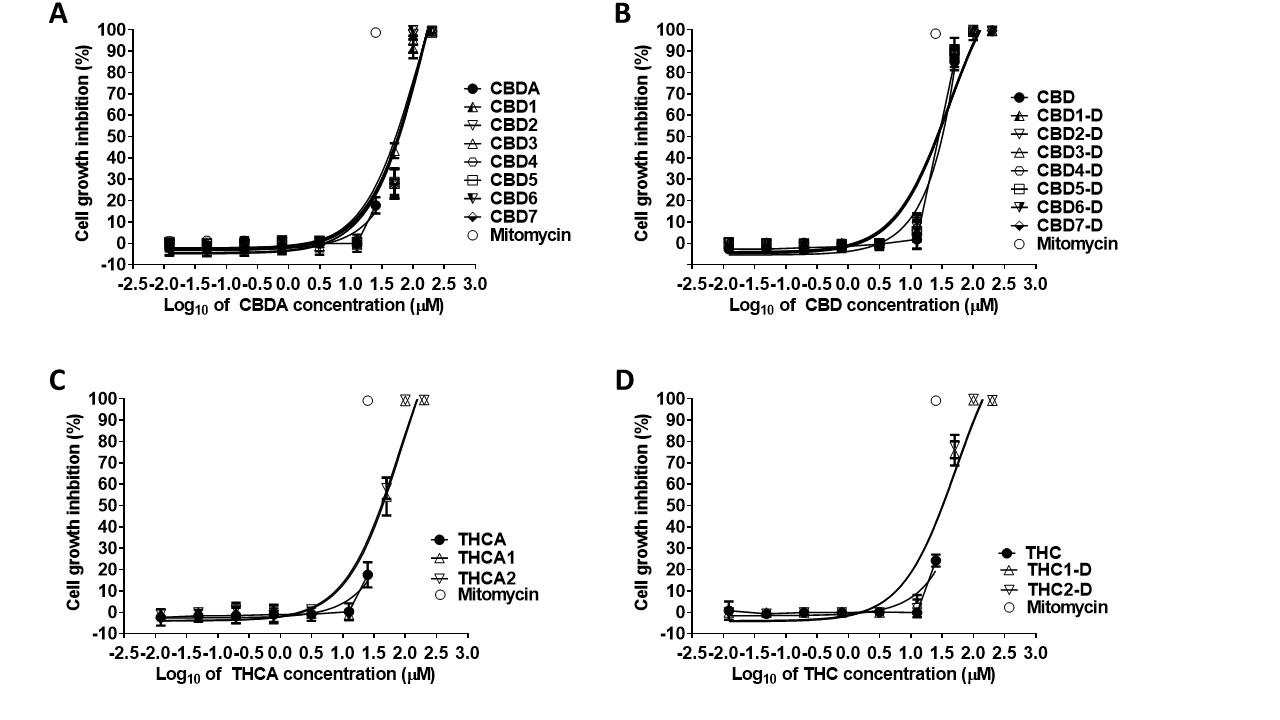


**Supplementary Figure 1.** Cytotoxicity of non-decarboxylated and carboxylated extracts of CBD and THC *C. sativa* genotypes against Vero 6 cells. A. CBDA denotes cannabidiolic acid while CBDA1, CBDA2, CBDA3, CBDA4, CBDA5, CBDA6 and CBDA7 denote non-decarboxylated cannabidiolic acid extracts of Elleta, Harlequin, Strawberry, Mandarin, Lemon Heaven, Lemon Master and Fantasy Bud CBD genotypes, respectively. B. CBD denotes cannabidiol while CBD1-D, CBD2-D, CBD3-D, CBD4-D, CBD5-D, CBD6-D and CBD7-D denote decarboxylated cannabidiol extracts of Elleta Harlequin, Strawberry, Mandarin, Lemon Heaven, Lemon Master and Fantasy Bud CBD genotypes respectively. C. THCA denotes ∆9- tetrahydrocannabinolic acid while THCA-1 and THCA-2 denote non-decarboxylated ∆9-9tetrahydrocannabinolic acid extracts of Kosher Haze and Prima Holandica THC genotypes, respectively. D. THC denotes ∆9-tetrahydrocannabinol while THC1-D and THC2-D denote non-decarboxylated cannabidiol extracts of Kosher Haze and Prima Holandica THC genotypes, respectively. All extracts were tested in a 200.00-0.012 μM concentration range. Standard reference control compounds, CBDA, CBD, THCA and THC were tested in a concentration range 25.00-0.012 μM. Mitomycin at 10.00 μM concentration was used as the reference cytostatic drug. The inhibition of cellular growth of Vero 6 cells were determined in 72 h incubation assays. Data points represent the means (±SD) of three independent biological replicates with two technical replicates.
